# Impairment of Hepatic Growth Hormone and Glucocorticoid Receptor Signaling Causes Steatosis and Hepatocellular Carcinoma in Mice

**DOI:** 10.1002/hep.24509

**Published:** 2011-10

**Authors:** Kristina M Mueller, Jan-Wilhelm Kornfeld, Katrin Friedbichler, Leander Blaas, Gerda Egger, Harald Esterbauer, Peter Hasselblatt, Michaela Schlederer, Susanne Haindl, Kay-Uwe Wagner, David Engblom, Guenter Haemmerle, Dagmar Kratky, Veronika Sexl, Lukas Kenner, Andrey V Kozlov, Luigi Terracciano, Rudolf Zechner, Guenther Schuetz, Emilio Casanova, J Andrew Pospisilik, Markus H Heim, Richard Moriggl

**Affiliations:** 1 Ludwig-Boltzmann-Institute for Cancer ResearchVienna, Austria; 2 Institute for Genetics, Department of Mouse Genetics and Metabolism, University of CologneCologne, Germany; 3 Clinical Institute of Pathology, Medical University of ViennaVienna, Austria; 4 Department of Laboratory Medicine, Medical University ViennaVienna, Austria; 5 Department of Medicine II, Freiburg University HospitalFreiburg, Germany; 6 Ludwig-Boltzmann-Institute for Experimental and Clinical TraumatologyVienna, Austria; 7 Eppley Institute for Research in Cancer and Allied Diseases and the Department of Pathology and Microbiology, University of Nebraska Medical CenterOmaha, NE, USA; 8 Department of Clinical and Experimental Medicine, Faculty of Health Sciences, Linkoeping UniversityLinkoeping, Sweden; 9 Institute of Molecular BiosciencesGraz, Austria; 10 Institute of Molecular Biology and Biochemistry, Center of Molecular Medicine, Medical University of GrazGraz, Austria; 11 Institute of Pharmacology and Toxicology, Veterinary University of ViennaVienna, Austria; 12 Institute of Pathology, University Hospital BaselBasel, Switzerland; 13 German Cancer Research CenterHeidelberg, Germany; 14 Max-Planck-Institute of ImmunobiologyFreiburg, Germany; 15 Department of Biomedicine, Division of Gastroenterology and Hepatology, University Hospital BaselBasel, Switzerland

## Abstract

Growth hormone (GH)-activated signal transducer and activator of transcription 5 (STAT5) and the glucocorticoid (GC)-responsive glucocorticoid receptor (GR) are important signal integrators in the liver during metabolic and physiologic stress. Their deregulation has been implicated in the development of metabolic liver diseases, such as steatosis and progression to fibrosis. Using liver-specific STAT5 and GR knockout mice, we addressed their role in metabolism and liver cancer onset. STAT5 single and STAT5/GR double mutants developed steatosis, but only double-mutant mice progressed to liver cancer. Mechanistically, STAT5 deficiency led to the up-regulation of prolipogenic sterol regulatory element binding protein 1 (SREBP-1) and peroxisome proliferator activated receptor gamma (PPAR-γ) signaling. Combined loss of STAT5/GR resulted in GH resistance and hypercortisolism. The combination of both induced expression of adipose tissue lipases, adipose tissue lipid mobilization, and lipid flux to the liver, thereby aggravating STAT5-dependent steatosis. The metabolic dysfunctions in STAT5/GR compound knockout animals led to the development of hepatic dysplasia at 9 months of age. At 12 months, 35% of STAT5/GR-deficient livers harbored dysplastic nodules and ∼60% hepatocellular carcinomas (HCCs). HCC development was associated with GH and insulin resistance, enhanced tumor necrosis factor alpha (TNF-α) expression, high reactive oxygen species levels, and augmented liver and DNA damage parameters. Moreover, activation of the c-Jun N-terminal kinase 1 (JNK1) and STAT3 was prominent.

**Conclusion:**

Hepatic STAT5/GR signaling is crucial for the maintenance of systemic lipid homeostasis. Impairment of both signaling cascades causes severe metabolic liver disease and promotes spontaneous hepatic tumorigenesis. (hepatology 2011;54:1398–1409)

Hepatic steatosis is estimated to affect >20% of the Western population, with raising incidence partly caused by excess nutrition and a lack of exercise.^1^ Steatosis as a hallmark of nonalcoholic fatty liver disease (NAFLD) is connected to obesity, insulin resistance, and type II diabetes.^2^ A strong correlation between steatosis and insulin resistance has been demonstrated in human patients and animal models of NAFLD.^1^, ^3^-^6^ Persistent hepatic lipid accumulation contributes to chronic inflammation with progression to nonalcoholic steatohepatitis (NASH), cirrhosis, and hepatocellular carcinoma (HCC).^7^ Steatosis results from excessive free fatty acid (FFA) synthesis relative to oxidative clearance^8^, ^9^ and/or elevated lipid hydrolysis in adipose tissues. FA synthesis, clearance, and release are, among others, regulated by neuroendocrine factors, such as growth hormone (GH) or glucocorticoids (GCs), whose levels vary under conditions of changing energy supply. Both signaling pathways have been implicated in the development of NAFLD and metabolic syndrome.^10^, ^11^ Animal studies have revealed that the transcription of distinct signal transducer and activator of transcription 5 (STAT5) target-gene subsets requires cofactor function of the glucocorticoid receptor (GR).^12^, ^13^ The interaction of STAT5 and GR ensures the proper transcription of genes implicated in postnatal body growth, such as insulin-like growth factor-1 (IGF-1).^12^, ^13^ As serum IGF-1 levels negatively regulate the release of GH in the pituitary, an impairment of this autoinhibitory GH/STAT5/IGF-1 feedback loop leads to GH resistance. This is of clinical interest, because it is tightly associated with metabolic syndrome.^14^ Mice lacking STAT5 or the GH receptor (GHR) in the liver acquire characteristics of GH resistance and develop steatosis and insulin resistance.^3^, ^4^ Importantly, hepatic STAT5 deficiency contributes to CCl_4_−induced liver fibrosis and HCC development.^15^ Furthermore, hepatocyte-specific deletion of JAK2 also results in GH resistance and the development of hepatic steatosis. However, these mice harbor no defects in glucose and insulin homeostasis.^16^

We aimed to investigate whether the regulation of hepatic lipid homeostasis (1) requires synergism of STAT5 and GR signaling or (2) both signaling cascades affect lipid metabolism independently. We confirm previous findings^3^, ^4^, ^17^ that STAT5 deficiency causes steatosis, insulin resistance, and glucose intolerance. However, the combined deletion of hepatic STAT5 and GR led to severe fatty liver disease resulting from a combination of hepatic GH resistance and hypercortisolism. The former resulted from the liver-specific ablation of STAT5, and the latter was from the deletion of the GR in hepatocytes. A combination of both conditions, as found in compound STAT5/GR mutants, induced peripheral lipodystrophy, additional liver lipid accumulation, and, subsequently, tumorigenic transformation of hepatocytes.

## Materials and Methods

### Mice

Mice with a hepatic deletion of STAT5 and/or the GR were generated as described.^13^ Littermates not expressing Alfp-*Cre* recombinase served as controls. For experimental procedures, we used male mice, if not stated otherwise. Mice were kept at the Decentralized Biomedical Facilities, Medical University of Vienna (Vienna, Austria), under standardized conditions. All animal experiments were carried out according to an ethical animal license protocol, and our contract was approved by university and Austrian Ministry authorities.

### Western Blotting

Liver homogenates were prepared as previously described.^13^ Blots were incubated with antibodies against STAT5b (rabbit polyclonal antibody, epitope aa775-788), pY-STAT5 (#71-6900; Invitrogen, Carlsbad, CA), heat shock cognate 70-kDa protein (HSC-70) (sc-7298; Santa Cruz Biotechnology, Santa Cruz, CA), GR (sc-1004; Santa Cruz Biotechnology), and antibodies against total levels and the phosphorylated isoforms of p38, extracellular signal-regulated kinases 1 and 2 (ERK1/2), and c-Jun N-terminal kinases 1 and 2 (JNK1/2) (mitogen-activated protein kinase [MAPK] Sampler Kits #9926 and #9910; Cell Signaling, Beverly, MA).

### Other Materials and Methods

Animal and histology procedures, quantitative reverse-transcription polymerase chain reaction (qRT-PCR), serum biochemistry, determination of hepatic triglyceride levels, immunohistochemistry, and the measurement of reactive oxygen species (ROS) levels are described in the Supporting Materials and Methods.

### Statistical Analyses

Results are presented as mean ± standard error of the mean. Statistical analyses were performed by analysis of variance, followed by Dunn's or Tukey's post-hoc tests. Data were considered statistically significant (*P < 0.05; **P < 0.01; ***P < 0.001).

## Results

### Deletion of Hepatic STAT5 and the GR Causes Steatosis and Lipodystrophy

**Fig. 1:**
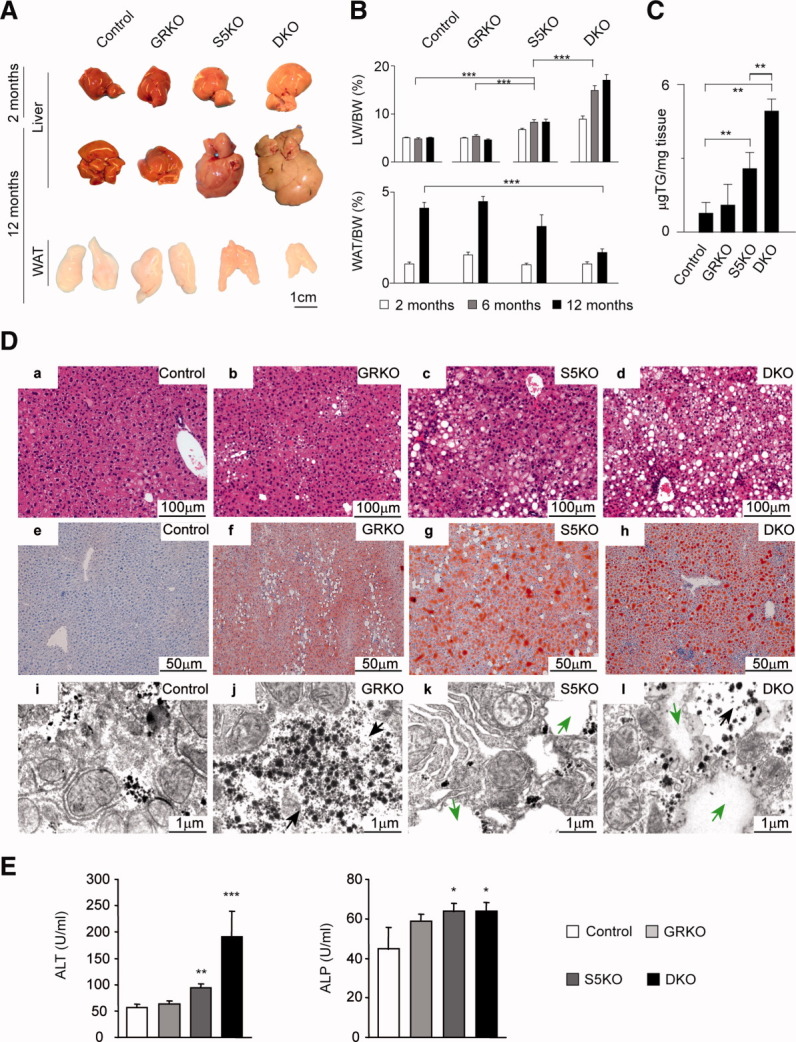
DKO mice develop severe steatosis, hepatomegaly, and lipodystrophy. (A) Macroscopic appearance of livers and epigonadal WAT in mutant and control mice at indicated time points. (B) Liver weight (LW)/body weight (BW) and WAT/BW ratios of mutant and control mice at indicated time points (n = 8/genotype/time point). (C) Hepatic triglyceride content in 2-month-old mice (n = 5/genotype). (D) Liver histology of livers from 6-month-old mice. (a-d) Liver sections were stained with hematoxylin and eosin. (e-h) Lipid accumulation in livers was visualized by Oil Red O on cryosections. (i-l) Electron microscopy analysis of fat distribution in livers of 2-month-old mice (cytoplasmic lipid droplets, green arrows; intrahepatic glycogen granules, black arrows). (E) Serum liver-damage parameters ALT and ALP of 2-month-old mice (n ≥ 5/genotype). *P < 0.05; **P < 0.01; ***P < 0.001.

To investigate whether hepatic lipid homeostasis would require STAT5-GR synergism or whether the two transcription factors would affect lipid metabolism independently, we conditionally deleted the GR (GRKO), STAT5 (S5KO), or STAT5 and the GR (double knockout [DKO]) in hepatocytes. Efficient deletion was confirmed by western blotting analyses (Supporting Fig. 1A). Macroscopic hepatomegaly and steatosis were first evident in 2-month-old S5KO and DKO mutants, as compared to GRKO and control mice. Although hepatomegaly in S5KO mutants remained stable, DKO mice displayed progressive fatty liver disease, with a 4-fold increase in liver mass by 12 months of age (Fig. 1A, B) and an 8-fold rise in hepatic triglyceride (TG) content as early as 2 months of age (Fig. 1C). Strikingly, a dramatic depletion of white adipose tissue (WAT) was observed exclusively in DKO mice (−58%; Fig. 1A,B). Histological examination revealed a significantly increased mean score of steatosis in young (83% versus 49%), but not in aged, DKO mice, compared to age-matched S5KO mutants (77% versus 53%; Supporting Fig. 1B). Micro- and macrovesicular steatosis in S5KO (Fig. 1D, c, g, and k) and DKO mice (Fig. 1D, d, h, and l) was associated with elevated serum levels of alanine aminotransferase (ALT) and alkaline phosphatase (ALP) as indicators of liver injury (Fig. 1E). In contrast, histological analysis revealed a normal liver architecture in control (Fig. 1D, a, e, and i) and GRKO animals (Fig. 1D, b, f, and j) at all time points analyzed. A summary of the histological analysis is given in Supporting Table 1. Taken together, the STAT5-dependent fatty degeneration of hepatocytes is severely aggravated upon additional GR deletion in the liver.

### STAT5 Regulates Hepatic De Novo Lipogenesis Independently of GR Cofactor Interaction

Hyperglycemia, hyperinsulinemia, and elevated resistin levels in both STAT5-deficient lines suggested hepatic insulin resistance upon STAT5 loss (Supporting Fig. 2A). Oral-glucose and insulin-tolerance tests confirmed insulin resistance and glucose intolerance^3^ (Supporting Fig. 2B). At the molecular level, defects in insulin receptor (IR) signaling, such as reduced tyrosine phosphorylation of the IR, IR substrates 1 and 2 (IRS-1 and -2), and serine phosphorylation of AKT were evident in both STAT5-deficient lines upon insulin administration (Supporting Fig. 2C). We detected increased transcript and protein levels of sterol regulatory element binding protein 1 (SREBP-1) and peroxisome proliferator-activated receptor gamma (PPAR-γ) in *S5KO* and *DKO* livers. In line with this, the gene expression of SREBP-1 (*Fasn* and *Scd2*) and PPAR-γ targets (*Dgat1, Dgat2*, and *Cd36*) was found to be increased. Transcript levels of *Fgf21*, which negatively control SREBP-1 maturation and activation, were decreased in single-knockout and *DKO* livers. Furthermore, messenger RNA (mRNA) and protein levels of lipogenic CCAAT enhancer binding protein (C/EBP)α and C/EBPβ were elevated (Supporting Fig. 3A,B; Supporting Table 2). Using chromatin immunoprecipitation analysis, a significantly enriched binding of GH-activated STAT5 to the *Srebp-1a* and *Srebp-1c* promoter was observed (Supporting Fig. 3C,D). Furthermore, GH-induced STAT5 activation led to a marked, time-dependent decrease of *Srebp-1a* and *Srebp-1c* mRNA levels in control livers (Supporting Fig. 3E).

**Fig. 2:**
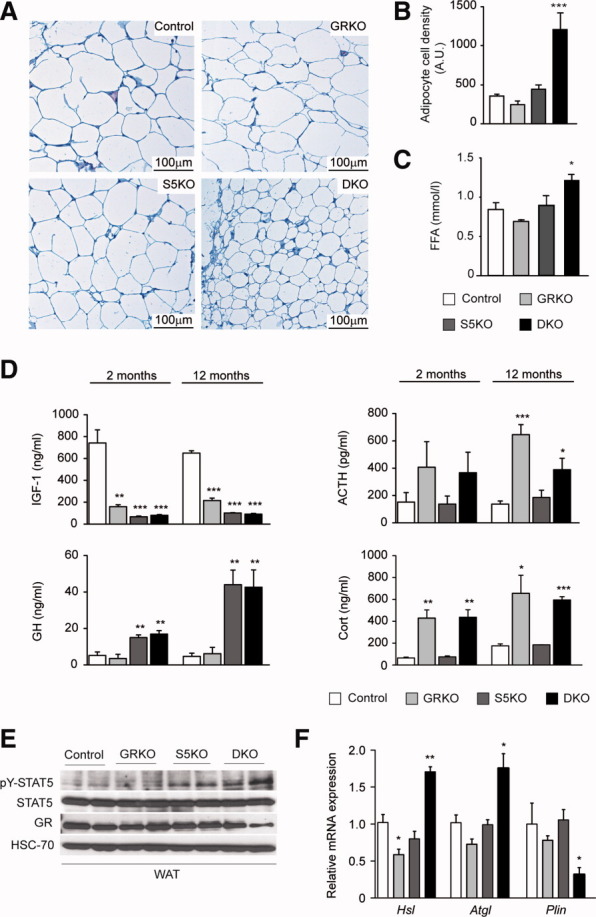
Combination of hepatic GH resistance and hypercortisolism causes peripheral lipodystrophy in DKO animals. (A) Histology of WAT. Epigonadal WAT of 12-month-old mice was stained with hematoxylin and eosin. (B) Quantification of WAT cell density. Hematoxylin and eosin–stained sections were used to analyze cell density using HistoQuest image analysis (TissueGnostics GmbH, Vienna, Austria). (C) Levels of FFA were determined in 2-month-old mice using colorimetric assays. (D) Levels of GH and IGF-1 were determined by ELISA. Levels of corticosterone (Cort) and ACTH were determined by radioimmunoassay (n = 8/genotype/time point). (E) Representative western blotting analysis of WAT homogenates from 2-month-old mice. Expression levels of STAT5 and GR proteins were determined using specific antibodies. HSC-70 served as the loading control. (F) Relative mRNA levels of *Hsl*, *Atgl*, and *Plin* were quantified by qRT-PCR in WAT from 6-month-old mice. Ct values were normalized to GAPDH (ΔCt method; n = 4/genotype). *P < 0.05; **P < 0.01; ***P < 0.001.

**Fig. 3:**
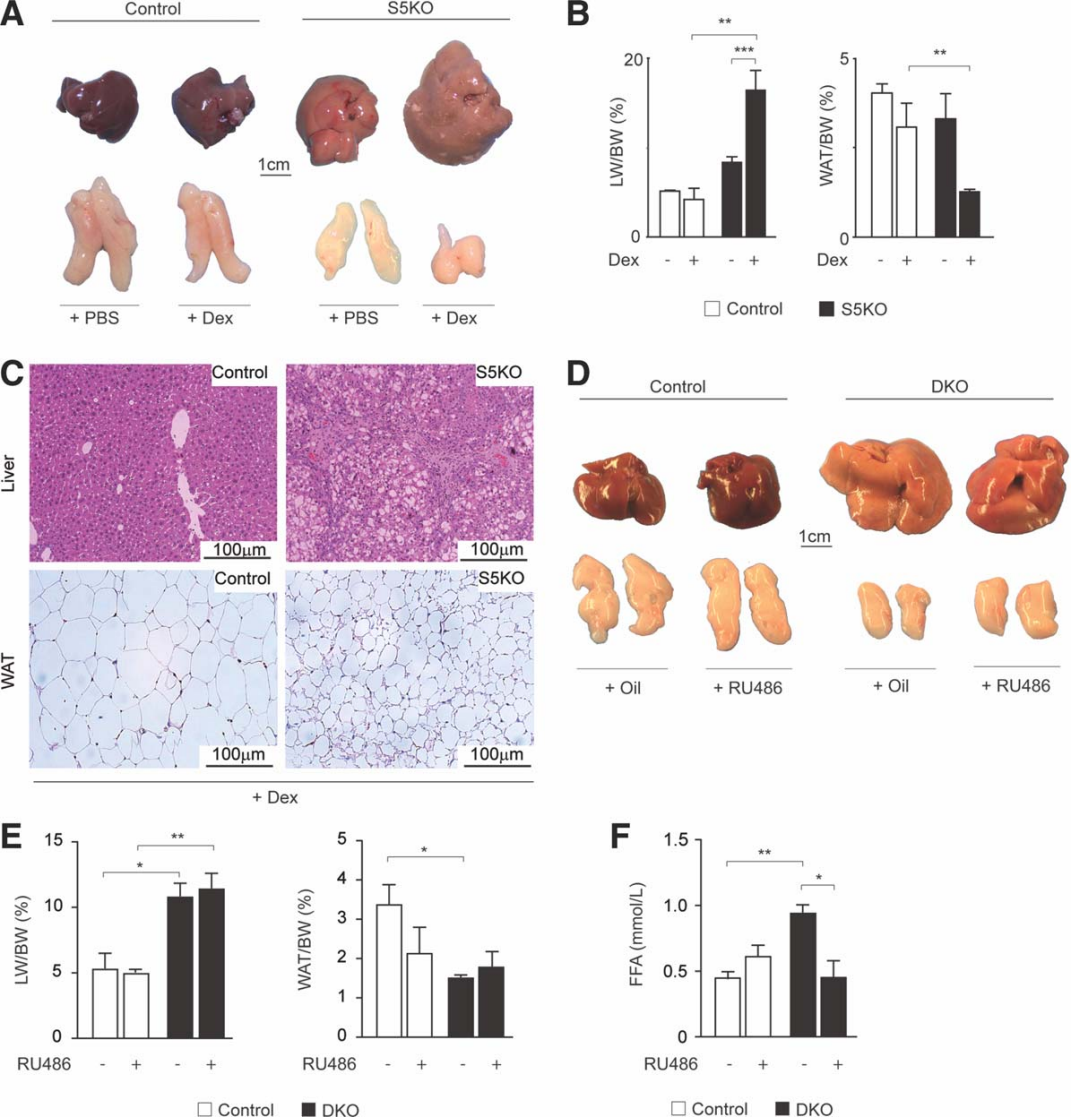
Impact of GR agonist or antagonist treatment on WAT lipolysis. (A) Macroscopic appearance of livers and WAT from S5KO mice following 14 days of dexamethasone (Dex) or mock treatment (phosphate-buffered saline). (B) LW/BW (left) and WAT/BW (middle) ratios of 6-month-old mutant mice of indicated genotypes and treatment. (C) Histological analysis of liver and WAT using hematoxylin and eosin–stained sections from control and S5KO mice after Dex treatment. (D) Macroscopic appearance of livers and WAT from DKO mice after 14 days of RU486 or mock (Oil) treatment. (E) LW/BW (left) and WAT/BW (middle) ratios of 6-month-old mice of indicated genotypes and treatment. (F) Levels of FFA were determined after RU486 or mock treatment of control and DKO mice using a colorimetric assay. For Dex and RU486 treatment: n ≥ 4/genotype/treatment. *P < 0.05; **P < 0.01; ***P < 0.001.

### Hepatic GH Resistance and Hypercortisolism Triggers Lipolysis of Adipose Tissue in DKO Mice

Phenotypically, DKO mice display an aggravation of liver phenotype, compared to S5KO mice. One explanation for the increased hepatic TG load in DKO might be alterations in whole-body lipid homeostasis, resulting in enhanced lipolysis of adipocytes and elevated hepatic FFA delivery (Fig. 1A,B). Analysis of epigonadal WAT, brown adipose tissue, and subcutaneous fat from DKO mice revealed a severe reduction in fat depots and adipocyte cell size, compared with control and single-mutant mice (Fig. 2A,B and data not shown). Accordingly, elevated levels of circulating FFA were found in DKO mice (Fig. 2C). As expected, both STAT5-deficient lines showed high serum levels of GH secondary to the loss of negative IGF-1 regulation^3^ (Fig. 2D, left row). Yet, unexpectedly, GRKO and DKO mutants developed hypercortisolism, that is, elevated serum levels of corticosterone and its positive regulator, adrenocorticotropic hormone (ACTH; Fig. 2D, right row). In line with this, adipocytes from DKO mice showed increased STAT5 and GR activation (Fig. 2E and data not shown). It was demonstrated that the simultaneous activation of GH-STAT5 and GC-GR signaling stimulates lipolysis in human adipocytes.^18^ Therefore, we quantified the transcript levels of major WAT lipases, that is, adipose triglyceride lipase (*Atgl*) and hormone-sensitive lipase (*Hsl*). We observed a significant up-regulation of *Atgl* and *Hsl* transcripts accompanied by the reduced gene expression of Perilipin (*Plin*), a major coating protein of adipocytes, exclusively in DKO WAT (Fig. 2F). To confirm that synergistic adipose GH/STAT5/GC-GR activation accounted for the induction of lipases and concomitant lipolysis in DKO mice, we pharmacologically mimicked the combination of GH resistance and hypercortisolism. Therefore, we administered the GR agonist, dexamethasone (Dex), to 6-month-old S5KO mice for 14 days. Although Dex treatment had no effect in control animals, Dex treatment severely aggravated hepatomegaly and steatosis in S5KO livers, accompanied by a decrease in WAT size (Fig. 3A,B). Histological analysis further confirmed the aggravation of hepatic steatosis and lipodystrophy in Dex-treated S5KO mice (Fig. 3C). To determine whether systemic GR inhibition would protect from increased WAT lipolysis in DKO mutants, we treated 6-month-old DKO mice with the GR antagonist, RU486, for 14 days. Macroscopically, no considerable changes in liver and WAT size of RU486-treated DKO, compared to control, animals could be observed (Fig. 3D,E). Yet, RU486 treatment of DKO mice normalized the amount of serum FFA to levels comparable to RU486- and vehicle-treated control mice (Fig. 3F). Thus, the combination of hepatic GH resistance and hypercortisolism in DKO mice results in a generalized depletion of adipose stores, which, in turn, aggravates the STAT5-dependent fatty liver phenotype.

### Spontaneous Development of Hepatocellular Carcinomas in DKO Mice

**Fig. 4:**
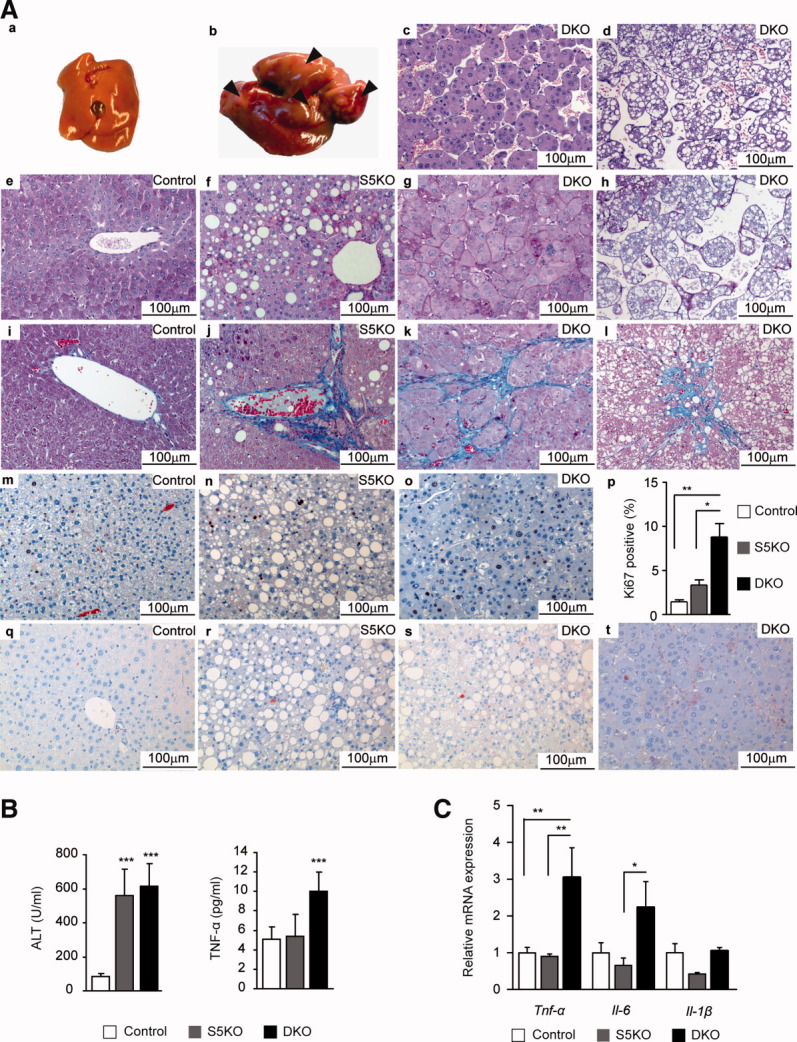
Spontaneous development of liver tumors in DKO mice. (A) Hepatocellular carcinoma (HCC) formation in 12-month-old DKO mice. (a) Control liver. (b) Macroscopic view of representative DKO liver. Arrows indicate tumors and atypical nodules. Representative hematoxylin and eosin–stained sections showing two different types of HCCs as either (c) solid and nonfatty or as (d) tumors containing lipid droplets. (e-h) Representative PAS staining for glycogen deposition. (i-l) Representative CAB staining for collagen deposition. (m-p) Quantification of Ki67-positive hepatocytes by immunohistochemistry showing enhanced proliferation of DKO livers. Ki67-positive hepatocytes were quantified using HistoQuest image analysis (n ≥ 5/genotype; TissueGnostics GmbH, Vienna, Austria). (q-t) Representative immunohistochemistry for cleaved caspase 3–positive hepatocytes showing no increase in apoptosis of DKO livers. (B) Serum liver-damage parameters ALT and TNF-α levels of 12-month-old mice (n ≥ 5/genotype). (C) Relative mRNA levels of proinflammatory cytokines were quantified by qRT-PCR in livers from 12-month-old mice and normalized to *Gapdh* (n = 6/genotype). *P < 0.05; **P < 0.01; ***P < 0.001.

Despite negligible fibrotic changes and mild inflammatory infiltration (Supporting Table 1), we observed the development of spontaneous liver tumors in DKO mice. Macroscopical examination of livers from 9-month-old DKO mice revealed atypical nodules (in 2 of 5), whereas S5KO and control mice showed no evidence of hepatic tumorigenesis at all time points analyzed. Furthermore, detailed phenotypic examination revealed no significant differences between control and GRKO mice (data not shown). Histological analysis of atypical nodules displayed distinct dysplastic lesions with vacuoles of accumulated fat compressing adjacent parenchyma (Supporting Fig. 4A, a-d). At 12 months of age, 35% (6 of 17) of DKO mice displayed dysplastic nodules and 59% (10 of 17) HCCs (Supporting Fig. 4B; Fig. 4A, a-b). Histological examination revealed well to moderately differentiated HCCs either of a (1) nonfatty and solid or (2) lipid-laden-tumor type, both of which displayed nuclear poly- and pleomorphism. Malignant hepatocytes were either growing in solid sheets or tended to aggregate in disorganized laminae (Fig. 4A, c-d; Supporting Fig. 4A, e-f). Periodic acid Schiff (PAS) staining revealed no significant necrotic degeneration of hepatocytes. Interestingly, solid/nonfatty tumors, in particular, displayed increased fibrous, pericellular collagen depositions, as illustrated by Chromotrop Anilinblue (CAB) staining (Fig. 4A, e-h and i-l). Hepatocyte proliferation was enhanced in DKO livers (∼8%), compared to S5KO (∼3%) and control livers (∼1%), as demonstrated by elevated numbers of Ki67-positive hepatocytes (Fig. 4A, m-p). However, no change in apoptotic rates of DKO livers was observed (Fig. 4A, q-t), and gene-expression levels of apoptosis regulating *Bcl-2* family members *Bcl-2*, *Bcl-x*_*L*_, and *Bax* were only slightly changed (Supporting Fig. 4C). At the time point analyzed, ALT levels were similarly increased in DKO and S5KO mutants, indicating potent hepatocyte damage in both groups. Next, we determined serum levels of the proinflammatory and tumor-promoting cytokines, tumor necrosis factor alpha (TNF-α) and interleukin (IL)-6. TNF-α was strongly elevated in DKO mice, whereas IL-6 levels were unchanged (Fig. 4B and data not shown). On the transcriptional level, we observed a strong up-regulation of *Tnf*-α and, to a lesser degree, *Il-6* mRNA in DKO livers, whereas hepatic *Il-1*β transcript levels were unchanged (Fig. 4C). Collectively, these data suggest that aggravation of the STAT5-dependent fatty liver phenotype caused by the additional deposition of extrahepatic lipids facilitates liver tumorigenesis.

### Mechanistic Insights in Tumor Development in DKO Mice

**Fig. 5:**
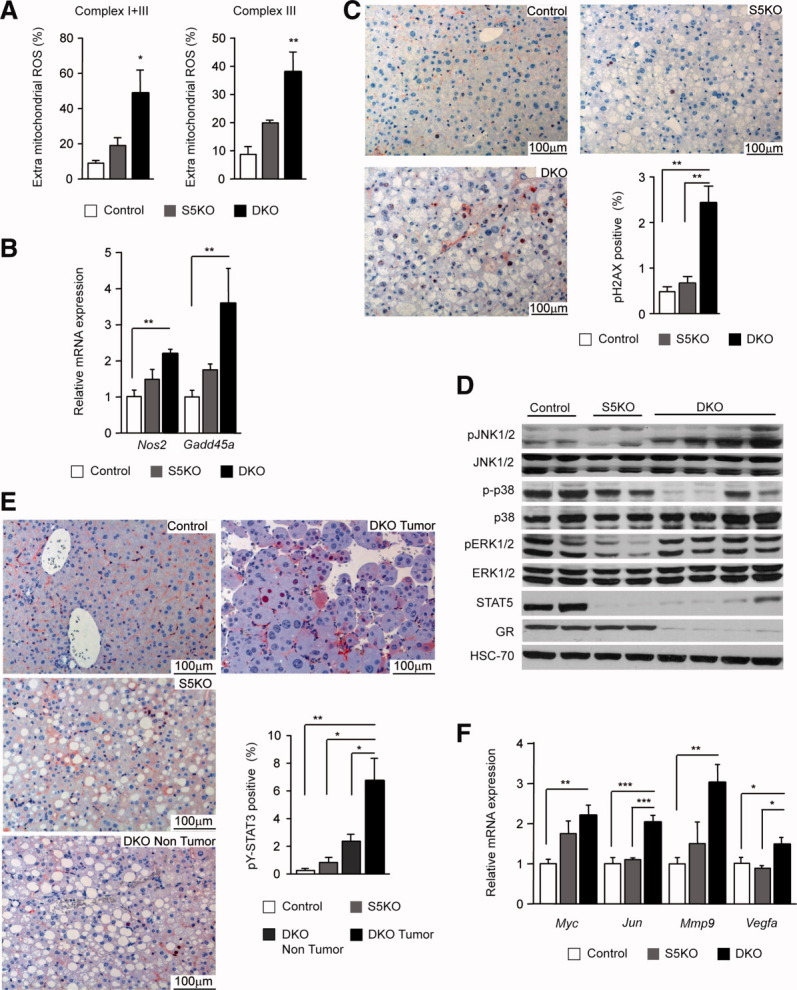
Oxidative stress-dependent hepatocyte damage and tumor-promoting signaling in DKO livers. (A) Extramitochondrial ROS production. ROS was determined using the 1-hydroxy-3-carboxy-pyrrolidine spin-trap method (n = 4/genotype). (B) Relative mRNA levels of *Nos2* and *Gadd45a* were quantified by qRT-PCR in livers from 12-month-old mice and normalized to *Gapdh* (n = 6/genotype). (C) DNA damage in DKO mice. Liver sections were stained with antibodies against phoshorylated H2AX. Positive hepatocytes were quantified using image analysis (n ≥ 5/genotype). (D) Representative western blotting analysis showing protein expression and activation of JNK1/2, p38, and ERK1/2 in 12-month-old mice. HSC-70 served as the loading control. (E) STAT3 activation in DKO HCCs. Liver sections were stained with antibodies against phoshorylated STAT3. Positive hepatocytes were quantified in control, S5KO, and DKO nontumor and DKO tumor tissue using HistoQuest image analysis (n ≥ 5/genotype; TissueGnostics GmbH, Vienna, Austria). (F) Relative mRNA levels of *Myc*, *Jun*, *Mmp9*, and *Vegfa* were quantified by qRT-PCR in livers from 12-month-old mice and normalized to *Gapdh* (n = 6/genotype). *P < 0.05; **P < 0.01; ***P < 0.001.

Progressive fatty degeneration of hepatocytes is associated with oxidative stress and subsequent hepatocyte damage, a process shown to contribute to tumorigenesis.^19^ Global gene-expression and subsequent gene set enrichment analysis revealed deregulated expression levels of several antioxidant genes already in 2-month-old DKO animals (Supporting Fig. 4D). Thus, we measured ROS production and release in liver mitochondria. Extramitochondrial ROS levels in DKO livers were increased ∼4-fold over control and ∼2-fold over S5KO livers (Fig. 5A). The transcription of inducible nitric oxide synthase, *Nos2*, which leads to ROS and reactive nitrogen species generation, was also up-regulated in DKO livers. At this time point, transcript levels of the DNA damage-responsive gene, *Gadd45a*, were strongly elevated, whereas the expression levels of two major antioxidant genes, *Sod1* and *Sod2*, were unchanged (Fig. 5B; Supporting Fig. 4E). Consistent with observed oxidative stress, DKO livers showed increased DNA damage, compared to control and S5KO livers, as assessed by the emergence of phoshorylated histone residues (pH2AX; Fig. 5C). To gain molecular insight in the processes governing the malignant transformation of hepatocytes in DKO animals, we determined the activation of the major stress-dependent MAPK-signaling pathways. These are triggered by continuous liver damage and are known to be involved in the pathogenesis of HCC. Tumor-bearing DKO mice exhibited elevated levels of JNK1 activity in the liver, which was almost absent in control and S5KO hepatocytes. In contrast, activation of ERK1/2 was unchanged between DKO and control livers, whereas p38 activation was reduced in DKO animals (Fig. 5D; Supporting Fig. 4F). HCCs displayed a modest increase in STAT3 phosphorylation, which was recently linked to hepatic tumorigenesis in the setting of chronic liver disease.^15^, ^20^ In nontumor liver tissue, however, STAT3 activity was almost not detectable (Fig. 5E). On the transcriptional level, livers from tumor-bearing mice exhibited significant up-regulation of *Myc*, *Jun*, *Mmp9*, and *Vegfa* that might contribute to an increased incidence of tumorigenesis (Fig. 5F). In summary, the development of HCCs in DKO mice coincides with oxidative stress and the activation of tumor-promoting JNK1- and STAT3-signaling cascades.

## Discussion

Hepatic GH- and GC-signaling cascades influence metabolic functions under conditions of altered energy balance and stress. Defects in either of the signaling pathways have been implicated in NAFLD development, including children with NAFLD progressing to end-stage liver disease.^10^, ^11^, ^21^-^23^ On the molecular level, steatosis is often associated with enhanced expression of the prolipogenic transcription factors, SREBP-1c and PPAR-γ. Recent studies have revealed an important role of STAT5 in the prevention of steatosis. This was partly linked to the observation that impairment of hepatic GH-STAT5 signaling causes enhanced gene expression of *Ppar*γ and its target gene, *Cd36*, which can also be directly regulated by STAT5.^3^, ^17^ Additionally, hepatic GHR deficiency resulted in enhanced *Srebp-1c* expression.^4^ Further studies have implicated SREBP-1c in *Ppar*γ transcription,^24^ whereas a bidirectional, inhibitory cross-talk between STAT5 and PPAR-γ was postulated.^25^ SREBP-1 is most likely activated in response to decreased expression of *Fgf21* and *Insig2*. Both transcripts are reported to be severely decreased upon liver-specific STAT5 deletion as well as upon systemic impairment of GHR signaling.^17^ Accordingly, we detected a decline in *Fgf21* mRNA levels in S5KO livers, which was found to be even more severe upon additional GR deficiency. However, the expression of liver X receptor (LXR) isoforms, which are known to regulate *Srebp-1* expression, was not significantly changed (data not shown). Additionally, we show that GH-activated STAT5 interacts with the promoter of both *Srebp-1* isoforms, which results in down-regulated expression. Taken together, these observations confirm a GH-STAT5-dependent regulation of hepatic lipogenesis on the transcriptional level, where STAT5 might repress *Srebp-1* isoforms. The induction of prolipogenic transcription factors and steatosis was not observed in GRKO livers, and it is also not present upon deletion of the N-terminal GR-interaction domain of STAT5 (as in STAT5^ΔN^ mice)^15^ (data not shown). Thus, we consider the induction of SREBP-1 and PPAR-γ-mediated lipogenesis as the primary effect of hepatic STAT5 deficiency. Moreover, hepatocyte-specific deletion of JAK2 causes massive steatosis and GH resistance. Interestingly, the phenotype was linked to increased peripheral GH-induced lipolysis and cluster of differentiation 36–mediated hepatic uptake of FFA, which could be rescued by the abrogation of GH secretion or partially normalized by antagonistic PPAR-γ action.^16^

Other conditions associated with steatosis are insulin resistance and glucose intolerance.^1^, ^6^ On the molecular level, insulin resistance is characterized by defects in IR signaling, which is observed upon the hepatocyte-specific deletion of the GH receptor or STAT5,^3^, ^4^ but not upon ablation of hepatic JAK2.^16^ Insulin resistance might be explained by observations made by others and similarly by us as follows. (1) A decrease in IRS-2-mediated signal transduction was accompanied by increased SREBP-1c associated with insulin resistance.^26^ Furthermore, when mice on a high-fat diet were treated with ezetimibe, a selective inhibitor of intestinal cholesterol absorption, down-regulation of hepatic SREBP-1c and reversed insulin resistance (IR) was a consequence, which was associated with increased pY-IRS-2 and pS-AKT.^27^ Similarly, we observed increased SREBP-1c mRNA and protein level in S5KO and DKO livers as well as impaired IR signal transduction. (2) The absence of insulin resistance in mice deficient for hepatic JAK2 might hint at a role of hepatic STAT5 in propagating IR signal transduction. It was shown that STAT5 is a physiological substrate of the IR *in vitro* and in tissues sensitive to insulin. Importantly, signaling through STAT5 upon insulin stimulation is JAK2 independent,^28^, ^29^ and insulin-stimulated STAT5 was shown to bind to the glucokinase promoter.^30^

Hepatic GH-STAT5 signaling influences GH/IGF-1 and insulin levels in the circulation,^3^, ^4^ whereas GR signaling counteracts the effects of high stress-hormone (e.g., corticosterone and ACTH) levels. GCs have been shown to suppress hepatic CBG (*Serpina6*) expression. Hence, GR knockout mice display high basal CBG levels that are not suppressed by Dexamethasone.^31^ Accordingly, we observed elevated *Serpina6* expression levels in GR-deficient livers, whereas expression of the GC-level regulating enzyme, 11β-HSD1, was unchanged (Supporting Fig. 5). This might suggest that upon hepatic GR deficiency, increased CBG expression results in elevated total serum GC levels. However, the increased CBG expression might lead to decreased unbound, active GCs and a subsequent decrease in negative feedback regulation, followed by enhanced ACTH and GC secretion.^32^, ^33^ Consistent with the notion of a strong induction of adipose tissue lipolysis by combined action of GH-STAT5 and GC-GR signaling, the enhanced shuttling of peripheral lipids to the liver was observed only in DKO mice. This process was associated with down-regulation of *Plin*^34^ and increased expression of *Hsl* and *Atgl*^35^, ^36^ in WAT, which triggered lipid mobilization from adipose tissue.

Recent studies revealed that hepatic GH-STAT5 signaling not only prevents steatosis, but also has protective functions in the context of genetically or chemically induced liver fibrosis and cancer development^15^, ^37^ (Friedbichler et al., unpublished). In addition, STAT5 counterbalances unscheduled cellular proliferation by inducing the cell-cycle inhibitors, *Cdkn2b* and *Cdkn1a*.^38^ This suggests that STAT5-deficient livers are more sensitive to hepatocyte damage and malignant transformation. Coupling the preexisting steatosis in STAT5-deficient livers with increased adipose tissue-derived lipid fluxes causes the spontaneous development of liver tumors in DKO mice. Other studies have applied genetic, chemical, and dietary-based liver insults to mimic chronic liver disease, and demonstrated that these conditions facilitate HCC development.^20^, ^39^, ^40^ However, the onset of HCCs in our model occurred in settings of progressive steatosis, despite minor inflammation and fibrosis. This was also reported in genetically obese mice, which develop spontaneous hepatic hyperplasia and harbor an age-dependent risk of HCC formation in the absence of apparent inflammation and fibrosis.^6^ Notably, also, in patients, the development of HCC is increasingly observed in the absence of advanced liver injury, with metabolic syndrome as the only identified risk factor.^41^ We suggest that tumorigenesis in DKO livers is a direct effect of the massive lipid accumulation causing persistent liver damage partly via increased mitochondrial ROS production and leakage. STAT5b deficiency is further associated with increased PPAR-α-dependent FFA oxidation in peroxisomes,^42^ which, possibly, contributes to additional ROS accumulation. It is well known that high ROS levels and concomitant DNA damage predisposes hepatocytes to malignant transformation. Oxidative stress and a subsequent vicious cycle of hepatocyte damage, apoptosis, and cellular replenishment were shown to contribute to liver tumorigenesis.^40^ However, as observed in the high-fat diet or obesity-induced liver cancer,^6^, ^20^ the increased ROS formation did not lead to enhanced hepatocyte apoptosis, whereas tumor-tissue proliferation was elevated. Excessive hepatic lipid accumulation and accompanying hepatocyte damage might activate tumor-promoting MAPK signaling during HCC development.^20^, ^43^ ERK1/2 and p38 MAPK signaling was not induced, whereas JNK1 activity was enhanced in tumor-bearing mice. Increase in FFA, TNF-α, and ROS levels (as observed during the onset and progression of NAFLD) are potent activators of JNK1 and are all found elevated/activated upon the development of murine and human HCC.^19^, ^20^, ^40^ Moreover, the activity of STAT3 that is frequently activated in human HCC and implicated in the development of chemically and obesity-induced HCC,^15^, ^20^, ^44^ was significantly enhanced in DKO tumors. The latter might be explained by (1) compensatory GH-dependent STAT3 activation under conditions of hepatic STAT5 deficiency^3^ (Supporting Fig. 6) and/or (2) elevated systemic and liver TNF-α levels in DKO mice, which can lead to IL-6 production^45^ and subsequent STAT3 activation. Finally, known tumor-promoting downstream effectors of *JNK1* and *STAT3* displayed enhanced expression (e.g., *Myc*, *Jun*, *Mmp9*, and *Vegfa*), which was restricted to *DKO* livers. In summary, our results underline the importance of hepatic GH-STAT5 and GC-GR signaling in the maintenance of systemic lipid homeostasis, where these pathways protect hepatocytes from metabolic stress and HCC development.

## Abbreviations

ACTH: adrenocorticotropic hormone
ALP: alkaline phosphatase
ALT: alanine aminotransferase
CBG: corticosteroid binding globulin
C/EBP: CCAAT enhancer binding protein
Dex: dexamethasone
DKO: double knockout
ERK1/2: extracellular signal-regulated kinases 1 and 2
FFA: free fatty acids
GCs: glucocorticoids
GH: growth hormone
GR: glucocorticoid receptor
HCC: hepatocellular carcinoma
HSC-70: heat shock cognate 70 kDa protein
IGF-1: insulin-like growth factor 1
IL: interleukin
iNOS: inducible nitric oxide synthase
IR: insulin receptor
IRS: insulin receptor substrate
JNK1/2: c-Jun N-terminal kinases 1 and 2
LXR: liver X receptor
MAPK: mitogen-activated protein kinase
mRNA: messenger RNA
NAFLD: nonalcoholic fatty liver disease
NASH: nonalcoholic steatohepatitis
Plin: perilipin
PPAR-γ: peroxisome proliferator-activated receptor gamma
qRT-PCR: quantitative reverse-transcription polymerase chain reaction
ROS: reactive oxygen species
STAT5: signal transducer and activator of transcription 5
SREBP-1: sterol regulatory element binding protein 1
TG: triglyceride
TNF-α: tumor necrosis factor alpha
WAT: white adipose tissue.
