# Supplementary material for: Impairment of Hepatic Growth Hormone and Glucocorticoid Receptor Signaling Causes Steatosis and Hepatocellular Carcinoma in Mice
Source: Hepatology. 2011 Oct;54(4):1398–409. doi: 10.1002/hep.24509 (PMC3232450; doi:10.1002/hep.24509)
Supplement: Supplementary file 1 [file hep0054-1398-SD1.doc]

**HEP-11-0409**

**Supporting Material and Methods**

**Mice**

Mice were fed a standard diet for nursing pups (Ssniff EF, R/M Kontrolle, Sniff GmbH, Soest, Germany). The nutrient composition as indicated by the manufacturer was: 22.1% raw protein, 4.5% raw fat, 46.8% starch and 10.8 % glucose.

**Quantitative Reverse Transcription Polymerase Chain Reaction**

**Figure 2 and Supporting Figure 3**

For quantitative reverse transcription polymerase chain reaction (qRT-PCR), one microgram of total RNA was reverse-transcribed into complementary DNA using a commercially available kit (Applied Biosystems, Carlsbad, CA). PCR products were obtained using gene-specific, intron-spanning primers. The SYBR green method was used for quantification of the PCR product and relative messenger RNA abundance was calculated using the ∆Ct-method. Messenger RNA (mRNA) levels were normalized to murine glyceraldehyde 3-phosphate dehydrogenase. Primer sequences are given in Supplementary Table 3.

**Figure 4, Figure 5 and Supporting Figure 4**

One microgram of total RNA was reverse-transcribed into complementary DNA using a commercially available kit (Applied Biosystems, Carlsbad, CA) and amplified using TaqMan Universal PCR Master Mix and NO AmpErase UNG with TaqMan Assay-on-Demand kits (Applied Biosystems). Relative expression of target mRNAs was determined using standard curves based on hepatocytes, and samples were adjusted for total RNA content by murine glyceraldehyde 3-phosphate dehydrogenase RNA levels by quantitative PCR. Calculations were performed by a comparative method (2∆∆*CT*). Quantitative PCR was performed on an ABI-Prism 7900 sequence detector (Applied Biosystems, Germany). Assays were linear over 4 orders of magnitude.

**Histology and Immunohistochemistry**

Tissues were removed quickly and fixed in 4% phosphate-buffered formalin for 48 hrs followed by embedding into paraffin using standard procedures. Sections (2-4 μm thick) were cut and stained with hematoxylin and eosine (HE), chromotrope aniline blue (CAB) and periodic acid-Schiff (PAS) using standard procedures. Hepatic lipids were visualized on cryosections (5 μm thick) by staining with the lipophilic Oil Red O dye (Sigma, Steinheim, Germany). For electron microscopy, mouse livers were cut into 1-2 mm thin pieces and fixed overnight in 1.6% glutaraldehyde. Quantification of adipose tissue cellularity in HE-stained sections was performed with the HistoQuest analysis software (TissueGnostics GmbH, Vienna, Austria; www.tissuegnostics.com) using a minimum of four fields of view. Briefly, the cell circumference and cell area were quantified and the ratio plotted on the y-axis as an indicator of adipocyte cell density in a given microscope field. High y-values represent a high adipocyte density. The x-axis values represent the intensity of hematoxylin staining of individual cells and were set arbitrarily to 1. Immunohistochemistry was performed on formalin-fixed, paraffin-embedded liver sections using the following antibodies: Ki67 (NCL-Ki67-P; Novocastra Laboratories, Newcastle, UK), pY-STAT5 (#9314), pY-STAT3 (#9145), pY-STAT1 (#9167), cleaved Caspase 3 (#9661), and pH2A.X (#9718, all from Cell Signaling, Beverly, MA). Quantification of positively stained hepatocytes was performed as described above.

**Mitochondrial Reactive Oxygen Species (ROS) Measurement**

Mitochondria were isolated from freshly harvested livers using standard procedures. The quantity of ROS was measured with Electron Paramagnetic Resonance (EPR) spectroscopy by incubating the isolated mitochondria with a spin trap and measuring the intensity of the signal. Isolated mitochondria were first mixed with an incubation buffer containing 80 mM potassium chloride, 5 mM potassium phosphate, 20 mM Tris-HCl, 1 mM DETAPAC and 0.1% BSA (pH 7.4). Either succinate [10.7 µM] or glutamate plus malate [10.7 µM] was added to stimulate respiration. A spin trap was then added to the mitochondria and the suspension incubated for 20 min on a shaking table at room temperature. After 20 minutes, a capillary tube filled with the suspension was inserted into the EPR and the spectrum is recorded. Final concentration of mitochondria was 1.0 mg/ml.

**Chromatin Immunoprecipitation**

Livers were harvested from untreated and GH injected control (2 mg/kg body weight, 30 min, n=4/group). After tissue homogenization, chromatin was cross-linked, isolated and sonicated. DNA-bound STAT5 was precipitated with a rabbit anti-STAT5 antibody (sc-835x; Santa Cruz Biotechnology, Santa Cruz, CA) pre-bound to magnetic beads (Dynabeads M280 Sheep anti-Rabbit IgG; Invitrogen, Carlsbad, CA). To exclude unspecific chromatin binding of the STAT5 antibody, we performed the ChIP in parallel with a rabbit-IgG antibody. Analysis of loci containing putative STAT5 binding sites was performed by qRT-PCR. Sequence conservation alignments between mouse (mm9), rat (rn4) and human (hg18) sequence annotations were performed using ECR browser (www.dcode.org) and TFSEARCH (www.cbrc.jp/research/db/TFSEARCH.html). Primer sequences are given in Supporting Table 3.

**Western Blot and Immunoprecipitation**

Liver homogenates were prepared as described (1). 40 µg of liver homogenate were subjected to SDS-PAGE, transferred to nitrocellulose membranes and incubated with antibodies against STAT5b (rabbit polyclonal antibody, epitope aa775-788), anti-total-AKT (sc-8312; Santa Cruz), anti-pS-AKT (#9271S; New England Biolabs, Ipswich, MA), C/EBPα (sc-061x), C/EBPβ (sc-150x), PPARγ (sc-7273), SREBP-1 (sc-13551), HSC-70 (sc-7298), GR (sc-1004; all from Santa Cruz Biotechnology, Santa Cruz, CA), pY-STAT5 (#71-6900, Invitrogen, Carlsbad, CA), pY-STAT3 (#9145) and pY-STAT1 (#9171, both from Cell Signaling, Beverly, MA). For immunoprecipitation, 1.5 mg of total protein were precipitated using antibodies against total-IRS-1 (#06248), total-IRS-2 (#06506, both Upstate Biotech., Lake Placid, NY) or the insulin receptor beta chain (IR-beta, sc-711, Santa Cruz) and probed with the anti-phosphotyrosine-specific antibody 4G10 (Millipore, Billerica, MA). The quantification of phospho-protein levels was performed by the ImageJ software (Rasband, W.S., ImageJ, U. S. National Institutes of Health, Bethesda, Maryland, USA, http://imagej.nih.gov/ij/, 1997-2011.) to compare the relative density of the respective phospho-protein bands normalized to the loading control HSC-70 (pJNK1 and pp38: n≥4/group; pY-STAT1: n=2/group).

**Serum Biochemistry and Animal Procedures**

Total lipids were extracted by the method of Folch (1) and dried in a stream of nitrogen and redissolved by brief sonication in 1% Triton X-100. Plasma TG and FFA levels were determined using colometric assays (ThermoElectron, Waltham, MA and Wako Chemicals, Neuss, Germany). Serum GH, IGF-1, TNF-α and Resistin concentrations were determined using ELISA kits (Millipore). Corticosterone and ACTH levels were quantified using RIA kits (MP Biochemicals, Solon, OH). Serum levels of alanine aminotransferase (ALT), alkaline phosphatase (AP), glucose and total cholesterol were measured using Reflotron system-based test-strips (Roche Applied Science, Mannheim, Germany).

For some experiments, mice were injected intraperitoneally with 2 mg/kg recombinant hGH (Immunotools, Friesoythe, Germany) and sacrificed at indicated time points.

Dexamethasone (20 µg/ml, Sigma, Steinheim, Germany) was administrated in the drinking water for 14 consecutive days. RU486 (20 mg/kg, Sigma, Steinheim, Germany) was dissolved in sunflower oil and given by intraperitoneal injections for 14 consecutive days. Control mice received daily oil injections. Mice were sacrificed by asphyxiation and blood was harvested by heart puncture. Specimens from liver and WAT were removed, snap-frozen in liquid nitrogen and stored at -80 °C for biochemical analysis.

Insulin tolerance test (ITT) was performed following a 4 h fast, oral glucose tolerance tests (OGTT) after an overnight fast. Glucose was orally administered at a final concentration of 1 g/kg, human insulin (0.75 U/kg; Novo Nordisk, Vienna, Austria) was injected intraperitoneally. Blood samples were taken from the tail vein at indicated time points and glucose levels determined using a glucometer (OneTouch Ultra, LifeScan, Vienna, Austria). Plasma insulin was determined using an enzyme-linked immunosorbent assay (ELISA; Crystal Chem Inc., Downers Grove, IL).

**Microarray Analysis**

We performed a specific analysis for genes involved in lipid metabolism and antioxidative defense on raw data from a recent study as described (2). Messenger RNA from three livers was pooled on each array, three arrays were used for each mutant group (S5KO and DKO) and nine arrays for the control group (litter-mates to the mutant mice). Arrays were normalized using GCRMA in affylmGUI. Significance levels were calculated in affylmGUI. In order to find molecular signatures in the data we looked for coordinated changes in functional gene-sets using MAPPFinder (3). We limited the analysis to around one hundred hand-picked, pre-defined groups, of which some where custom-made from papers describing profiles from the livers of mice lacking different transcription factors or administered with specific agonists or antagonists. Many lists were also taken from the GSEA software and consist of genes with certain motifs in their promoter regions as identified in a systematic study of regulatory motifs in mammals (4). The two criteria for inclusion of genes in the MAPPFinder-analysis were p<.001 or p<.05. This was done to pick up high magnitude changes of small groups of genes and low-amplitude changes in large groups, respectively. Gene-sets with p-value <.05 after correction for multiple comparisons were considered as significant.

**Supplementary References**

1. Folch J, Lees M, Sloane Stanley GH. A simple method for the isolation and purification of total lipides from animal tissues. J Biol Chem 1957;226:497-509.

2. Engblom D, Kornfeld JW, Schwake L, Tronche F, Reimann A, Beug H, et al. Direct glucocorticoid receptor-STAT5 interaction in hepatocytes controls body size and maturation-related gene expression. Genes Dev 2007;21:1157-1162.

3. Doniger SW, Salomonis N, Dahlquist KD, Vranizan K, Lawlor SC, Conklin BR. MAPPFinder: using Gene Ontology and GenMAPP to create a global gene-expression profile from microarray data. Genome Biol 2003;4:R7.

4. Xie X, Lu J, Kulbokas EJ, Golub TR, Mootha V, Lindblad-Toh K, et al. Systematic discovery of regulatory motifs in human promoters and 3' UTRs by comparison of several mammals. Nature 2005;434:338-345.
