# Supplementary figures and images for: Impairment of Hepatic Growth Hormone and Glucocorticoid Receptor Signaling Causes Steatosis and Hepatocellular Carcinoma in Mice
Source: Hepatology. 2011 Oct;54(4):1398–409. doi: 10.1002/hep.24509 (PMC3232450; doi:10.1002/hep.24509)

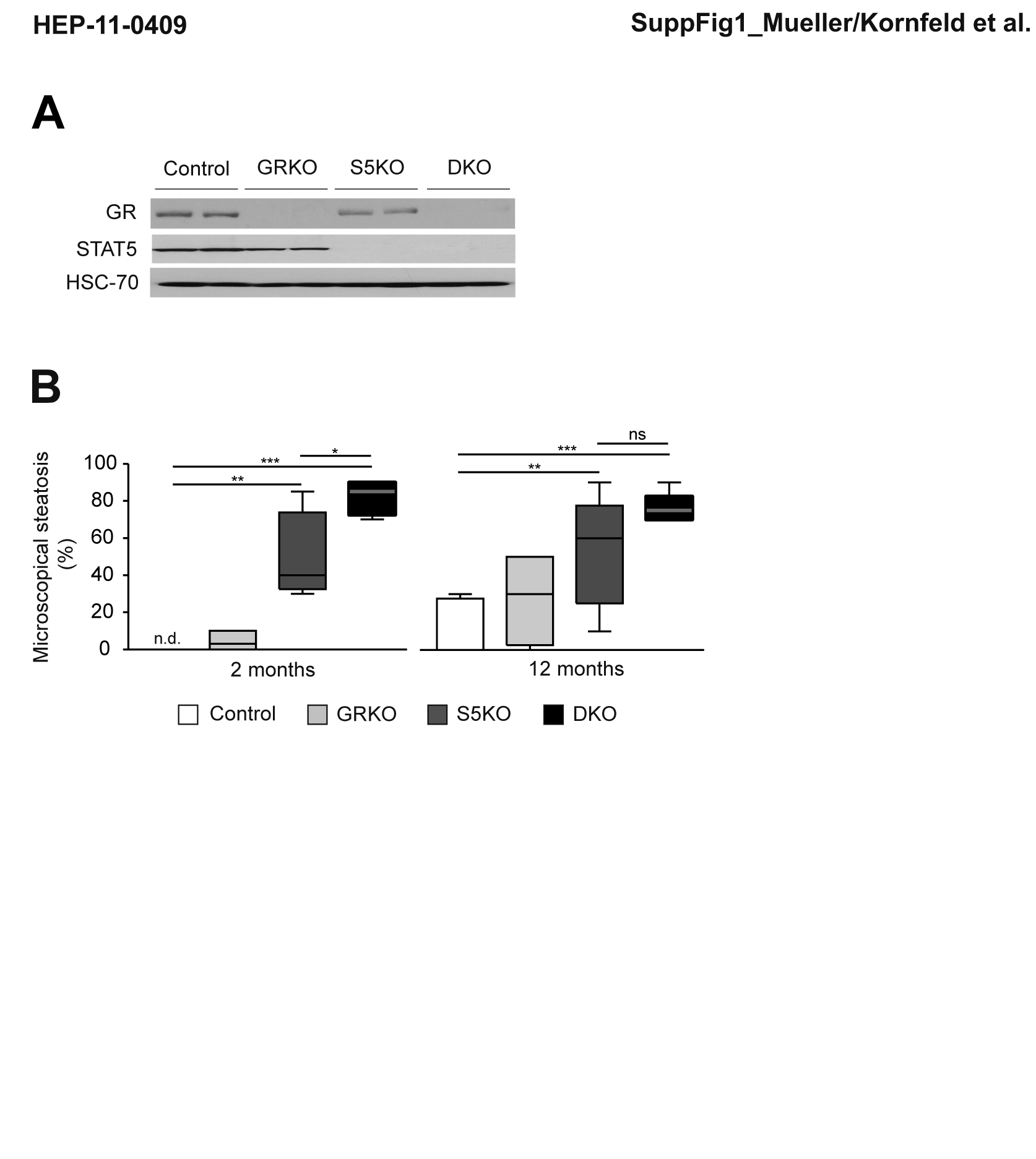

Supplement: Supplementary file 2 [file hep0054-1398-SD2.tif]

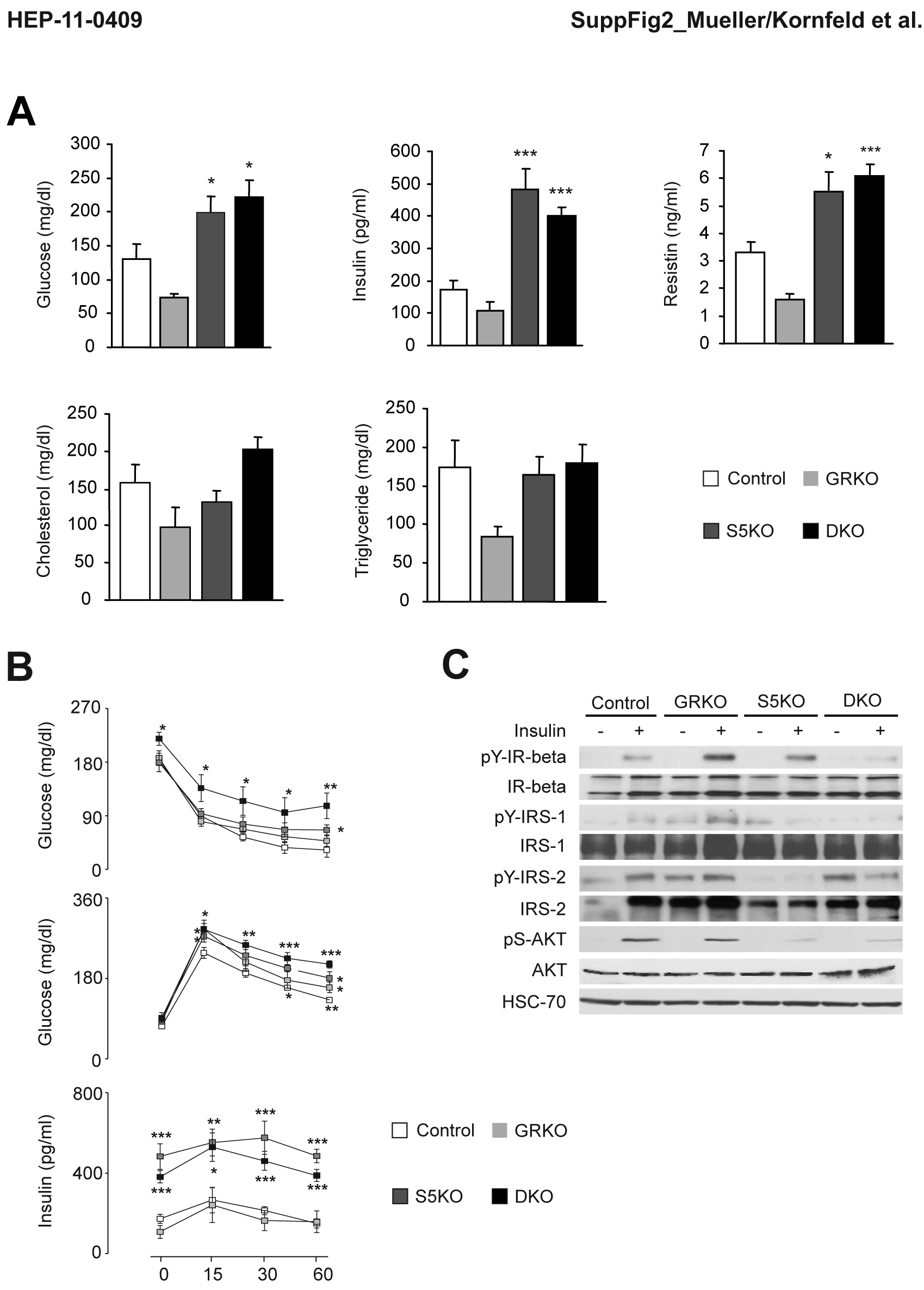

Supplement: Supplementary file 3 [file hep0054-1398-SD3.tif]

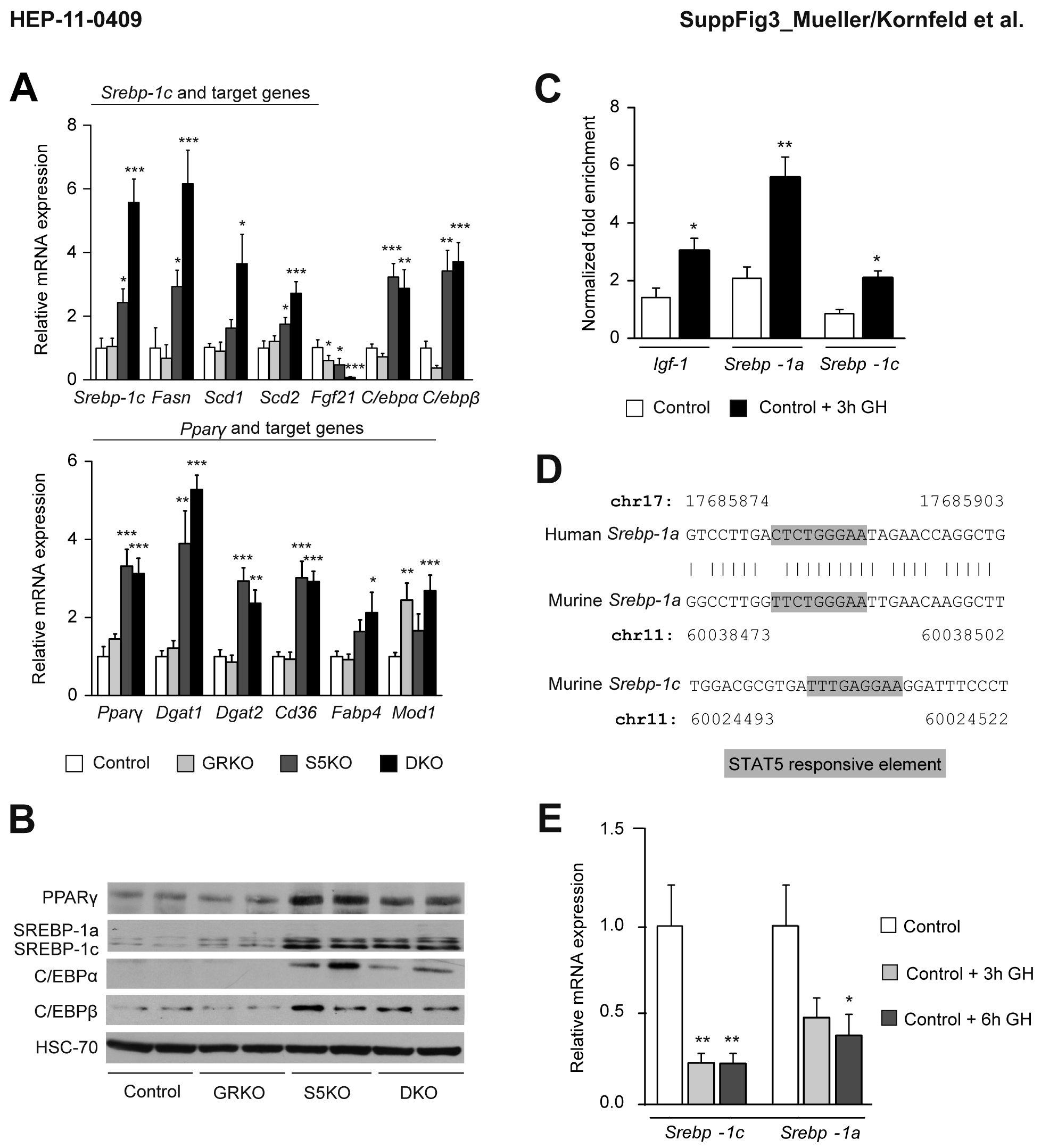

Supplement: Supplementary file 4 [file hep0054-1398-SD4.tif]

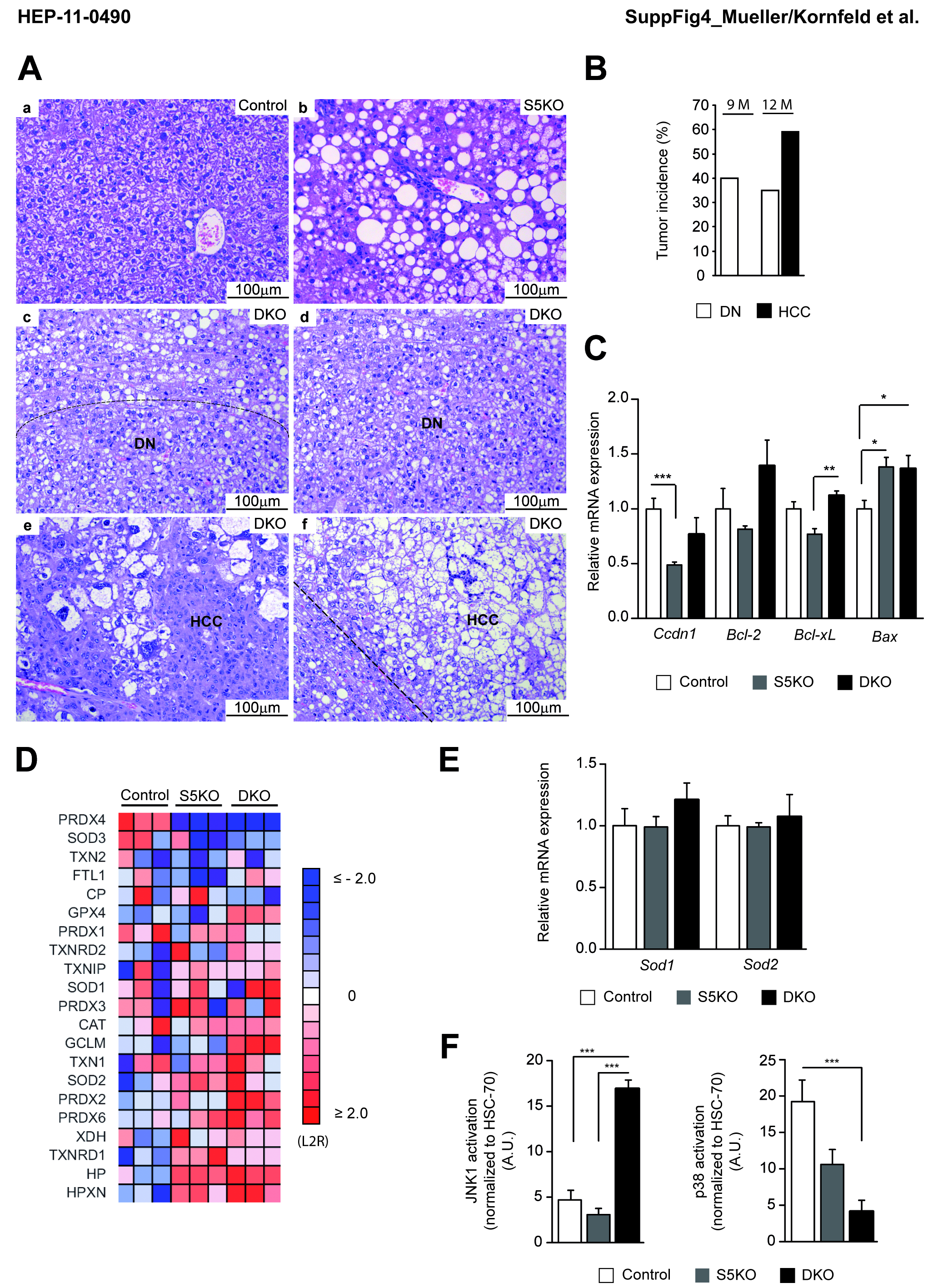

Supplement: Supplementary file 5 [file hep0054-1398-SD5.tif]

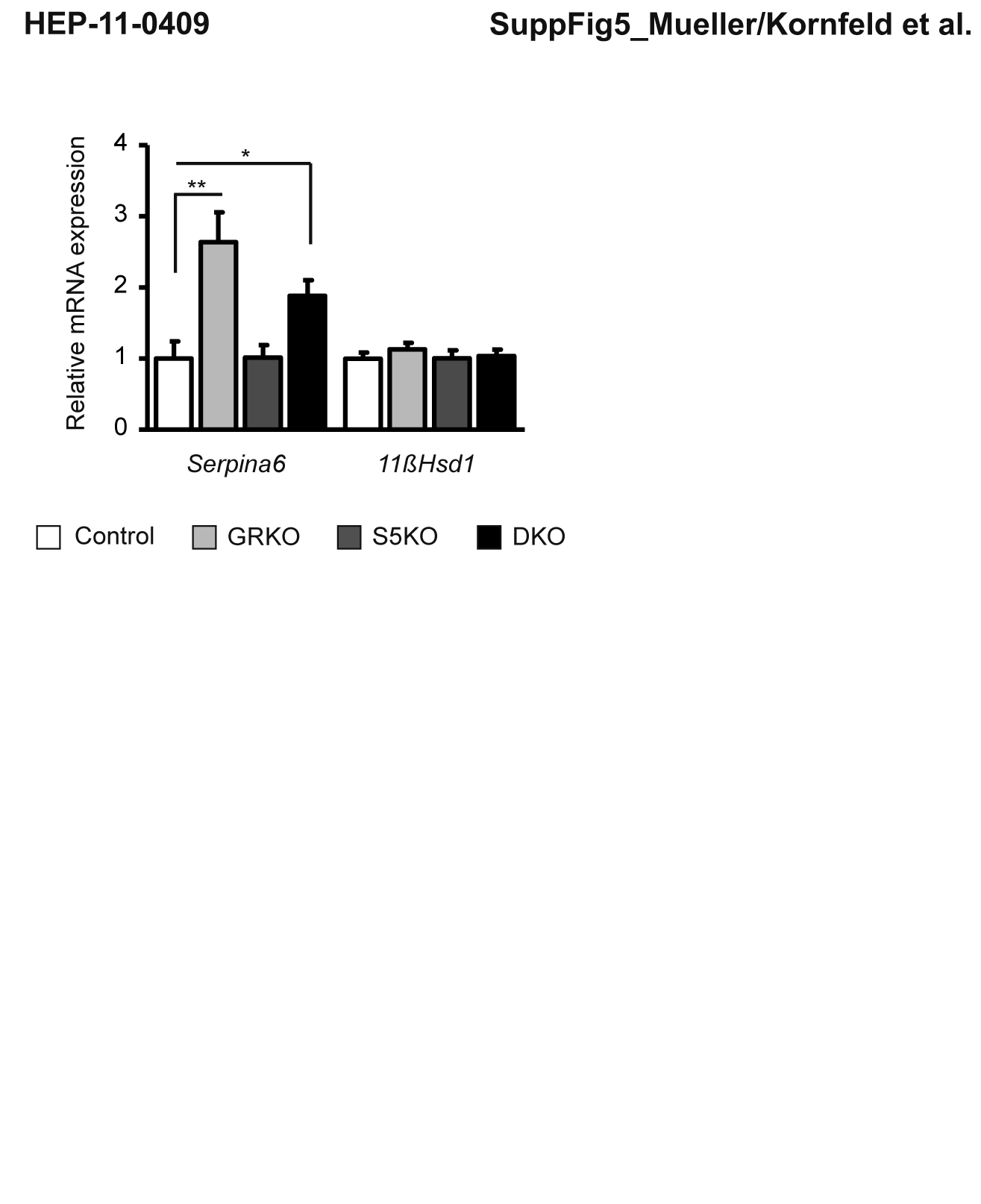

Supplement: Supplementary file 6 [file hep0054-1398-SD6.tif]

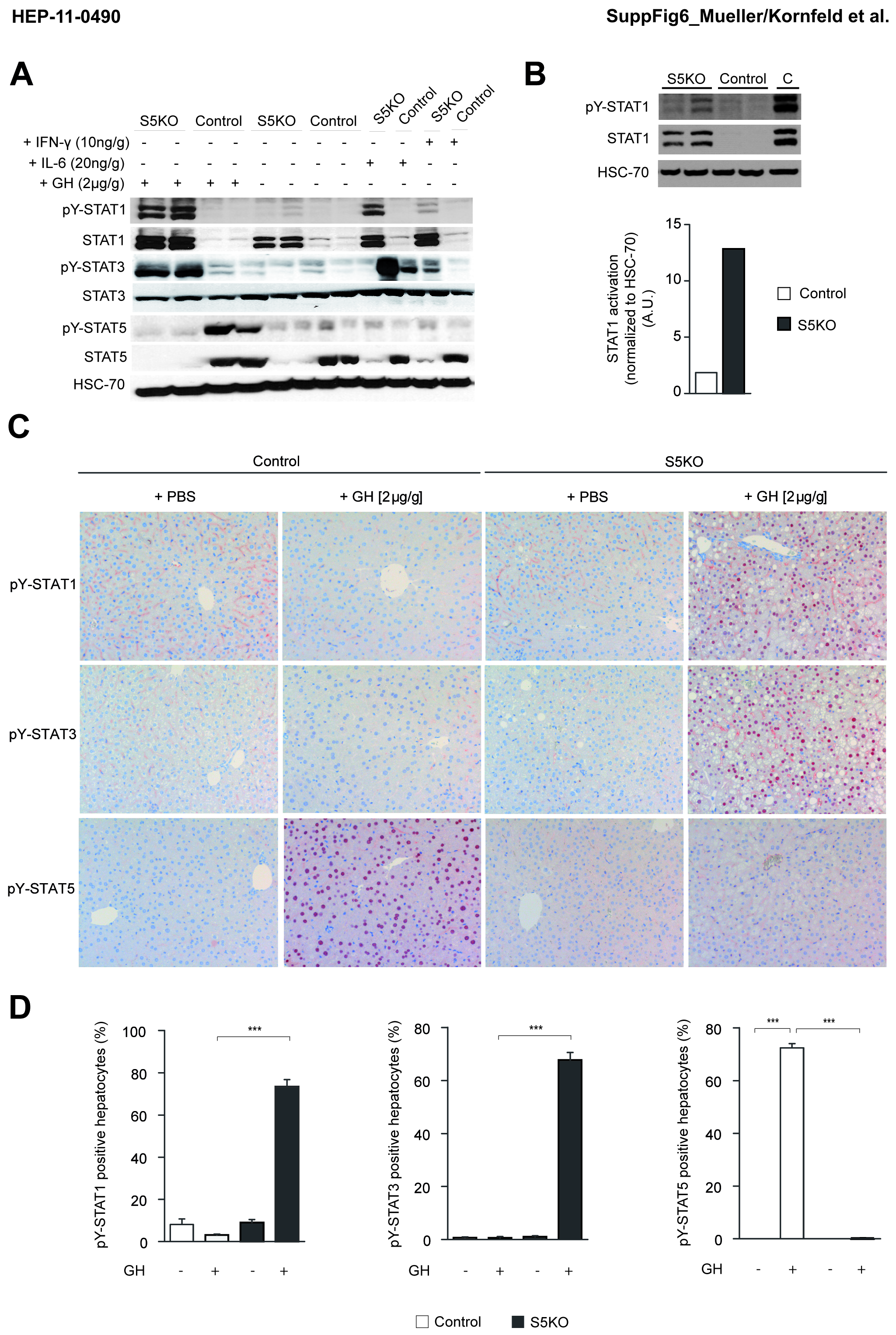

Supplement: Supplementary file 7 [file hep0054-1398-SD7.tif]
