# Supplementary material for: Impairment of Hepatic Growth Hormone and Glucocorticoid Receptor Signaling Causes Steatosis and Hepatocellular Carcinoma in Mice
Source: Hepatology. 2011 Oct;54(4):1398–409. doi: 10.1002/hep.24509 (PMC3232450; doi:10.1002/hep.24509)
Supplement: Supplementary file 8 [file hep0054-1398-SD8.doc]

**HEP-11-0409**

**Supporting Table 1. Detailed histology analysis of mice at indicated time-points.**

| Genotype | Age | Sex | Steatosis | Steatosis | Inflammatory | Ballooning | Fibrosis | Fibrosis |
| --- | --- | --- | --- | --- | --- | --- | --- | --- |
|  |  |  | [Type] | [%] | Infiltrates | Hepatocytes | [Lobular] | [Portal] |
|  |  |  |  |  |  |  |  |  |
| Control | 2 | m | 0 | 0 | 0 | 0 | 0 | 0 |
|  |  | m | 0 | 0 | 0 | 0 | 0 | 0 |
|  |  | m | 0 | 0 | 0 | 0 | 0 | 0 |
|  |  | m | 0 | 0 | 1 | 0 | 0 | 0 |
|  | 6 | m | 0 | 0 | lobular 1 | 3 | 0 | 0 |
|  |  | m | 0 | 0 | 0 | 0 | ND | ND |
|  |  | m | 0 | 0 | 0 | 0 | ND | ND |
|  |  | m | 0 | 0 | 0 | 0 | ND | ND |
|  |  | m | Micro | 5 | 0 | 0 | ND | ND |
|  |  | m | 0 | 0 | lobular 1 | 0 | 0 | 0 |
|  | 9 | m | Micro | 30 | 0 | 2 | PV 1 | 1 |
|  |  |  |  |  |  |  | PC 1 |  |
|  |  | m | 0 | 0 | lobular 1 | 0 | ND | ND |
|  |  | m | Micro | 30 | lobular 1 | 0 | ND | ND |
|  |  | m | 0 | 0 | lobular 1 | 0 | ND |  |
|  |  |  |  |  | portal 1 |  |  |  |
|  | 12 | m | 0 | 0 | 0 | 2 | 0 | 0 |
|  |  | m | 0 | 0 | 0 | ND | ND | ND |
|  |  | m | 0 | 0 | 0 | ND | ND | ND |
|  |  | m | 0 | 0 | lobular 1 | ND | ND | ND |
|  |  | m | 0 | 0 | lobular 1 | ND | ND | ND |
|  |  | m | Macro | 30 | lobular 1 | ND | ND | ND |
|  |  | m | Micro | 20 | lobular 1 | ND | ND | ND |
|  |  | m | Macro | 30 | lobular 1 | 0 | PV 1 | 1 |
|  |  |  |  |  | portal 1 |  | PC 1 |  |
|  |  |  |  |  |  |  |  |  |
| GRKO | 2 | m | Macro | 10 | 0 | 0 | 0 | 0 |
|  |  | m | 0 | 0 | 0 | 0 | 0 | 0 |
|  |  | m | Micro | 10 | 0 | ND | ND | ND |
|  |  | m | Micro | 3 | 0 | ND | 0 | 0 |
|  |  | m | 0 | 0 | 0 | ND | 0 | 0 |
|  | 6 | m | Macro | 30 | 0 | ND | ND | ND |
|  |  | m | Macro | 20 | lobular 1 | ND | ND | ND |
|  |  | m | Macro | 20 | lobular 1 | ND | ND | ND |
|  |  | m | Micro | 20 | 0 | ND | ND | ND |
|  | 9 | m | Micro | 30 | lobular 1 | ND | ND | ND |
|  |  | m | Micro | 40 | lobular 1 | ND | ND | ND |
|  |  | f | Macro | 25 | lobular 1 | 0 | PV 1 | 0 |
|  |  | f | Macro | 35 | lobular 1 | 3 | PV 1 | 1 |
|  |  |  |  |  | portal 1 |  |  |  |
|  | 12 | m | 0 | 0 | lobular 1 | ND | ND | ND |
|  |  | m | Micro/ | 50 | lobular 1 | ND | ND  ND | ND  ND |
|  |  |  | Macro |  |  |  |  |  |
|  |  | m | Micro | 10 | 0 | ND | ND | ND |
|  |  | m | Micro/ | 50 | 0 | ND | ND | ND |
|  |  |  | Macro |  |  |  |  |  |
|  |  |  |  |  |  |  |  |  |
| S5KO | 2 | m | Micro | 40 | 0 | 0 | PV 1 | 1 |
|  |  | m | Micro | 30 | lobular 1 | 0 | 0 | 0 |
|  |  | m | Micro | 40 | lobular 1 | 0 | 0 | 1 |
|  |  | m | Micro | 85 | lobular 1 | 0 | 0 | 0 |
|  | 6 | f | Micro/ | 80 | lobular 1 | 1 | PV 1 | 0 |
|  |  |  | Macro |  |  |  | PC 1 |  |
|  |  | f | Micro/ | 95 | lobular 1 | 1 | 0 | 0 |
|  |  |  | Macro |  | portal 1 |  |  |  |
|  |  | f | Micro/ | 40 | lobular 1 | 1 | PV 1 | 0 |
|  |  |  | Macro |  |  |  | PC 1 |  |
|  |  | f | Micro | 75 | 0 | 1 | 0 | 0 |
|  | 9 | f | Micro/ | 95 | lobular 1 | 2 | PV 1 | 0 |
|  |  |  | Macro |  | portal 1 |  |  |  |
|  |  | f | Micro/ | 75 | lobular 1 | 1 | PV 2 | 1 |
|  |  |  | Macro |  | portal 1 |  | PC 2 |  |
|  |  | m | Micro/ | 60 | lobular 1 | 1 | 0 | 0 |
|  |  |  | Macro |  |  |  |  |  |
|  |  | f | Macro | 50 | lobular 1 | 1 | PV 1 | 1 |
|  |  |  |  |  | portal 2 |  |  |  |
|  | 12 | f | Micro/ | 90 | lobular 1 | 1 | 0 | 0 |
|  |  |  | Macro |  | portal 1 |  |  |  |
|  |  | m | Micro/ | 65 | lobular 1 | 1 | PV 1 | 2 |
|  |  |  | Macro |  | portal 3 |  | PC 1 |  |
|  |  | m | Micro | 60 | lobular 1 | 0 | PC 1 | 1 |
|  |  |  | Macro |  | portal 1 |  |  |  |
|  |  | m | Micro | 40 | lobular 1 | 2 | PV 2 | 1 |
|  |  |  | Macro |  | portal 2 |  | PC 2 |  |
|  |  | f | Macro | 10 | 0 | 0 | PV 3 | 0 |
|  |  |  |  |  |  |  | PC 3 |  |
|  |  |  |  |  |  |  |  |  |
| DKO | 2 | f | Micro | 90 | lobular 1 | 0 | 0 | 0 |
|  |  | m | Micro | 70 | lobular 1 | 0 | 0 | 0 |
|  |  | m | Micro | 80 | 0 | 0 | 0 | 0 |
|  |  | f | Micro | 90 | 0 | 0 | ND |  |
|  | 6 | m | Micro | 90 | lobular 1 | 0 | PV 1 | 0 |
|  |  | f | Micro | 90 | lobular 2 | 2 | ND |  |
|  |  | m | Micro | 90 | lobular 1 | 1 | 0 | 0 |
|  |  |  |  |  | portal 1 |  |  |  |
|  | 9 | m | Micro/ | 90 | lobular 1 | 2 | PV 2 |  |
|  |  |  | Macro |  |  |  | PC 2 |  |
|  |  | m | Macro | 80 | lobular 2 | 0 | 0 | 0 |
|  |  | m | Micro/ | 90 | lobular 1 | 1 | PV 2 | 0 |
|  |  |  | Macro |  | portal 1 |  | PC 2 |  |
|  |  | f | Micro/ | 90 | lobular 3 | 2 | PV 2 | 1 |
|  |  |  | Macro |  | portal 2 |  | PC 1 |  |
|  | 12 | m | Micro/ | 90 | Lobular 1 | 1 | PV 2 | 0 |
|  |  |  | Macro |  |  |  | PC 2 |  |
|  |  | m | Micro/ | 70 | lobular 1 | 1 | PV 3 | 0 |
|  |  |  | Macro |  | portal 1 |  | PC 3 |  |
|  |  | m | Micro/ | 80 | lobular 1 | 1 | PV 2 | 0 |
|  |  |  | Macro |  |  |  | PC 2 |  |
|  |  | f | Macro | 80 | lobular 1 | 1 | 0 | 0 |
|  |  |  |  |  | portal 1 |  |  |  |
|  |  | m | Micro/ | 70 | lobular 1 | 0 | 0 | 0 |
|  |  |  | Macro |  |  |  |  |  |
|  |  | m | Macro | 70 | lobular 1 | 0 | 0 | 0 |
|  |  |  |  |  | portal 2 |  |  |  |

Micro, microvesicular; Macro, macrovesicular; ND, not determined; Pv, perivenular; Pc, pericellular
