# Supplementary material for: Impairment of Hepatic Growth Hormone and Glucocorticoid Receptor Signaling Causes Steatosis and Hepatocellular Carcinoma in Mice
Source: Hepatology. 2011 Oct;54(4):1398–409. doi: 10.1002/hep.24509 (PMC3232450; doi:10.1002/hep.24509)
Supplement: Supplementary file 9 [file hep0054-1398-SD9.doc]

**HEP-11-0409**

**Supporting** Table 2. Transcription factor signature analysis of genes statistically upregulated in Affymetrix expression profiling.

| **Symbol** | **Name** | **TF pathway** | **DKO** | | **S5KO** | | | | **GRKO** | | **Verif. (qPCR)** |
| --- | --- | --- | --- | --- | --- | --- | --- | --- | --- | --- | --- |
|  |  |  |  |  |  | |  | |  |  |  |
| **Lipid metabolism, lipogenic** | | **p=0.022**  **(for Pparγ)** | **x-fold** | **p-value** | **x-fold** | | **p-value** | | **x-fold** | **p-value** |  |
| *Pparγ* | PPARgamma |  | 1,77 | 0,004 | 1,68 | | 0,006 | | 1,38 | 0,071 | X |
| *Scd1* | stearoyl-Coenzyme A desaturase 1 | Pparγ/  Srebp-1c | 1,53 | 0,009 | 1,31 | | 0,076 | | 1,40 | 0,028 | X |
| *Scd2* | stearoyl-Coenzyme A desaturase 2 | Srebp-1c | 9,13 | 0,000 | 4,76 | | 0,000 | | 4,53 | 0,000 | X |
| *Lipe* | lipase, hormone sensitive | Pparγ | 1,46 | 0,000 | 1,19 | | 0,032 | | 1,14 | 0,100 |  |
| *Mod1* | malic enzyme, supernatant | Pparγ | 2,75 | 0,002 | 1,72 | | 0,061 | | 2,13 | 0,012 | X |
| *Fasn* | fatty acid synthase | Pparγ /  Srebp-1c | 1,56 | 0,032 | 1,18 | | 0,400 | | 1,65 | 0,018 | X |
| *Cd36* | CD36 antigen (fatty acid translocase) | Pparγ | 1,37 | 0,049 | 1,53 | | 0,010 | | 1,29 | 0,098 | X |
| *Fabp4* | fatty acid binding protein 4 adipocyte | Pparγ | 1,07 | 0,2906 | 0,98 | | 0,81 | | 0,98 | 0,81 | X |
| *Dgat1** | diacylglycerol O-acyltransferase 1 | Pparγ | 1,04 | 0,466 | 1,09 | | 0,250 | | 1,01 | 0,924 | X |
| **Lipid metabolism, others** | | | | | | | | | | | |
| *Lpin1* | lipin 1 |  | 0,08 | 0,000 | | 0,39 | 0,033 | 0,27 | | 0,005 |  |
| *Fabp5* | fatty acid binding protein 5, epidermal |  | 0,20 | 0,000 | | 0,08 | 0,000 | 0,37 | | 0,015 |  |
| *Apoa4* | apolipoprotein A4 |  | 0,22 | 0,007 | | 0,44 | 0,115 | 0,10 | | 0,000 |  |
| *Hdlbp* | (HDL binding protein |  | 0,73 | 0,002 | | 0,85 | 0,070 | 0,83 | | 0,037 |  |
| *Dbi* | diazepam binding inhibitor |  | 1,25 | 0,010 | | 1,04 | 0,564 | 1,23 | | 0,013 |  |
| *Apoc2* | apolipoprotein C2 |  | 1,88 | 0,001 | | 1,74 | 0,003 | 1,15 | | 0,400 |  |
| *Fto* | fatso |  | 0,63 | 0,008 | | 0,92 | 0,587 | 0,86 | | 0,324 |  |
| *Lipc* | lipase, hepatic |  | 0,69 | 0,006 | | 0,64 | 0,001 | 1,14 | | 0,259 |  |
| *Es* | esterase 31 |  | 0,33 | 0,004 | | 0,30 | 0,002 | 0,17 | | 0,000 |  |
| *Cebpβ* | CCAAT/enhancer binding protein, beta |  | 2,00 | 0,0446 | | 1,51 | 0,1964 | 0,56 | | 0,13 | X |
| *= not statistically significant in Affymetrix analysis, included for comparison | | | | | | | | | | | |
