# Supplementary material for: Impairment of Hepatic Growth Hormone and Glucocorticoid Receptor Signaling Causes Steatosis and Hepatocellular Carcinoma in Mice
Source: Hepatology. 2011 Oct;54(4):1398–409. doi: 10.1002/hep.24509 (PMC3232450; doi:10.1002/hep.24509)
Supplement: Supplementary file 10 [file hep0054-1398-SD10.doc]

**HEP-11-0409**

**Supporting Table 3. Primers used for qRT-PCR analysis.**

| **Application** | **Gene symbol** | **Accession No.** | **Sequence** |  |  |
| --- | --- | --- | --- | --- | --- |
| **qRT-PCR** | *Srebp-1a* | NM_011480 | for: CCGGGGAACTTTTCCTTAAC | | |
|  |  |  | rev: CTGTCTCACCCCCAGCATAG | | |
|  | *Srebp-1c* | NM_011480 | for: GCGTGGTTTCCAACATGACC | | |
|  |  |  | rev: CTCCAGCTCAGCTGTAGTGC | | |
|  | *Fasn* | NM_007988 | for: TGTCTGACACTGGCAATCTGAT | | |
|  |  |  | rev: CGGTCACACGGGTAGGTAGC | | |
|  | *Scd1* | NM_009127.3 | for: CGCTCTTTACCCTTTGCTG | | |
|  |  |  | rev: ATAGTCAGTTGCTCGCCTCAC | | |
|  | *Scd2* | NM_009128.1 | for: ATCTGCAGGATTGCCTCTGGG | | |
|  |  |  | rev: TCTCATGCTCACACCTACCCG | | |
|  | *Pparγ* | NM_011146.2 | for: ACCCAATGGTTGCTGATTAC | | |
|  |  |  | rev: CGGGAAGGACTTTATGTATGAG | | |
|  | *Ddat1* | NM_010046.2 | for: TGTGGTGATGCTGATCCTGAG | | |
|  |  |  | rev: GGATAGGATCCACCAGGATGC | | |
|  | *Dgat2* | NM_026384.3 | for: TGGCATAAGGCCCTATTTGG | | |
|  |  |  | rev: ATGGTGTCTCGGTTGACAGG | | |
|  | *Cd36* | NM_007643.3 | for: CCTTACTTGGGATTGGAGTGG | | |
|  |  |  | rev: CGGCTTTACCAAAGATGTAGCC | | |
|  | *Fabp4* | NM_024406.1 | for: TTCGATGAAATCACCGCAGA | | |
|  |  |  | rev: AGGGCCCCGCCATCT | | |
|  | *Mod1* | NM_008615.1 | for: AGTTGCTCTTGGGGTGGTGGC | | |
|  |  |  | rev: TAGTGCTGTACATCTGGGAGG | | |
|  | *Cebpα* | NM_007678 | for: ATGGAGTACAGCGGTGAGTATTC | | |
|  |  |  | rev: GTTGCCACTGGACATCTCTTC | | |
|  | *Cebpβ* | NM_009883.2 | for:GGTGGACAAGCTGAGCGACGAGTA | | |
|  |  |  | rev: AACAAGTTCCGCAGGGTGCTGAG | | |
|  | *Fgf21* | NM_020013.3 | for:GGAGGATGGAACAGTGGTAGG | | |
|  |  |  | rev:CAGTTCTCTGAAGCTGCAGGC | | |
|  | *Hsl* | NM_001039507.1 | for: TGAAGTGAGACCCATGGACGG | | |
|  |  |  | rev: GACCGAGTCATCTAGCATGGG | | |
|  | *Atgl* | NM_025802.2 | for: AGGGCCAAGAGGAAATTGGG | | |
|  |  |  | rev: TTCGTACCCAGTTGGGTAGGG | | |
|  | *Plin* | NM_175640.1 | for: ATGCCCTGAAGGGTGTTACGG | | |
|  |  |  | rev: TGTCTCGGAATTCGCTCTCGGG | | |
|  | *Serpina6* | NM_007618.2 | for: ACATCTGCCAGCACATCTTG | | |
|  |  |  | rev: TGTCGTCGCTGCACTTAATC | | |
|  | *Hsd11b1* | NM_001044751.1 | for: ATGACCCAGCCTATGATTGC | | |
|  |  |  | rev: GGAGAAGCTTGGGCGTTAAT | | |
| **ChIP** | *Srebp-1a* |  | for: CCGGGGAACTTTTCCTTAAC | | |
|  |  |  | rev: CTGTCTCACCCCCAGCATAG | | |
|  | *Srebp-1c* |  | for: GCGTGGTTTCCAACATGACC | | |
|  |  |  | rev: CTCCAGCTCAGCTGTAGTGC | | |
|  | *Igf-1* |  | for: CCCAAGCTGCAGAAGAGAAA | | |
|  |  |  | rev: TGATAACAGTATGCCAACACCA | | |
|  | *Cis* |  | for: TACCCCTTCCAACTCTGACTGAGC | | |
|  |  |  | rev: TTCCCTCCAGGATGTGACTGTG | | |
