# Supplementary material for: Impairment of Hepatic Growth Hormone and Glucocorticoid Receptor Signaling Causes Steatosis and Hepatocellular Carcinoma in Mice
Source: Hepatology. 2011 Oct;54(4):1398–409. doi: 10.1002/hep.24509 (PMC3232450; doi:10.1002/hep.24509)
Supplement: Supplementary file 11 [file hep0054-1398-SD11.doc]

**HEP-11-0409**

**Supporting Figure Legends**

**Supporting Figure 1.** Deletion of STAT5 and GR in hepatocytes and histological scoring of steatosis. (A) Representative Western blots of liver homogenates from 2-month-old male mice. Total levels of STAT5 and GR in Control and mutant livers were determined using specific antibodies. HSC-70 served as loading control. (B) The degree of histological steatosis in mutant livers at 2 and 12 months of age was scored semi-quantitatively by two board-certified liver pathologists (MH and LT). **P*<0.05; ***P* <0.01; ****P*<0.001.

**Supporting Figure 2.** Hepatic deletion of STAT5 using Alfp-*Cre* results in insulin resistance and impaired hepatic insulin receptor signaling. (A) Metabolic serum parameters of 2-month-old mice (n≥6/genotype). (B) Insulin Tolerance Tests (ITT): Insulin was administered through intraperitoneal injection following a 4h fast. Blood glucose levels were determined at given time points (upper panel). Oral Glucose Tolerance Tests (OGTT): Mice were fasted overnight and glucose was orally administered. Blood glucose levels were determined at given time points (middle panel). Plasma insulin levels following OGTT challenge. The insulin levels were determined by ELISA (lower panel). 2-month-old mice were analyzed for ITT and OGTT (n≥6/genotype). (B) Phosphorylation status of proteins implicated in insulin receptor downstream signaling following insulin challenge in 2-month-old mutant mice. Levels of phosphorylated and total protein were determined 15 min after intraperitoneal injection of insulin by immunoprecipitation and phospho-tyrosine/serine-specific immunoblot analysis. HSC-70 served as loading control. **P*<0.05; ***P* <0.01; ****P*<0.001.

**Supporting Figure 3.** Induction of PPARγ/SREBP-1 mediated lipogenesis upon hepatic STAT5 deficiency. (A) Relative mRNA levels of genes with functions in hepatic lipid metabolism were quantified by qRT-PCR in livers from 2-month-old mice. Ct values were normalized to *Gapdh* (n=6/genotype). (B) Representative Western blots showing protein expression of hepatic lipogenic transcription factors. HSC-70 served as loading control. (C) Chromatin immunoprecipitation (ChIP) demonstrating STAT5 binding to promoter regions of *Srebp-1a* and *Srebp-1c*. The STAT5 target gene *Igf-1* served as a positive control. Values are represented as fold induction versus a downstream region of *Cis* not containing STAT5 responsive elements. (D) Genomic localization of STAT5 responsive elements in the *Srebp-1a* and *Srebp-1c* promoter regions. The STAT5 responsive element in the *Srebp-1a* promoter is partially conserved between murine and humans. (E) Changes in relative hepatic *Srebp-1c* and *Srebp-1a* mRNA levels following GH administration for indicated durations (n=6/group). **P*<0.05; ***P* <0.01; ****P*<0.001.

**Supporting Figure 4.** Tumorigenesis in DKO mice. (A) Representative HE-stained sections of livers from 12-month-old Control and S5KO mice (a-b). Representative HE-stained sections of dysplastic nodules with vacuoles containing fat (c-d) and HCCs (e-f) observed in DKO livers. (B) Tumor incidence in 9-month-old (9 M) and 12-month-old (12 M) DKO mice. (C) Relative mRNA levels of *Ccdn1* and apoptosis regulating *Bcl-2* family members were quantified by qRT-PCR in livers from 12-month-old mice. Normalized to *Gapdh* (n=6/genotype). (D) Gene set enrichment analysis of antioxidant genes using Affymetrix® expression profile analysis. (E) Relative mRNA levels of antioxidant genes *Sod1* and *Sod2* were quantified by qRT-PCR in livers from 12-month-old mice. Normalized to *Gapdh* (n=6/genotype). (F) Quantification of JNK1 and p38 activation in livers from 12-month-old mice using image analysis (n≥4/genotype). **P*<0.05; ***P* <0.01; ****P*<0.001.

**Supporting Figure 5.** Relative mRNA expression levels of *Serpina6* and *11βHsd1* in livers from 2-month-old mice. Messenger RNA expression was quantified by qRT-PCR and Ct values were normalized to *Gapdh* (ΔCt method, n=6/genotype). **P*<0.05; ***P* <0.01.

**Supporting Figure 6.** Compensatory STAT1 and STAT3 activation upon hepatic deletion of STAT5 using Alfp-*Cre*. (A) Representative Western blots of liver homogenates from 2-month-old male mice. Amount of phosphorylation and total levels of STAT1, STAT3 and STAT5 in Control and S5KO livers either mock or growth hormone (GH) treated were determined using specific antibodies. HSC70 served as loading control. IFN-γ and IL-6 treated livers served as positive control. (B) Longer exposure of pY-STAT1 Western blot analysis of mock treated Control and S5KO livers and quantification of activated STAT1 using image analysis (n=2/group; C: IL-6 treated S5KO liver). (C) Immunohistochemistry of phoshorylated STAT1, STAT3 and STAT5 levels in Control and S5KO livers of 2-month-old male mice either mock or GH treated (30 min). (D) Quantification of activated STAT1, STAT3 and STAT5 levels in Control or S5KO livers upon mock or GH treatment using image analysis (n=3/genotype/treatment). **P*<0.05; ***P* <0.01; ****P*<0.001.
